# Supplementary material for: Elongator is a microtubule polymerase selective for polyglutamylated tubulin
Source: EMBO J. 2025 Jan 15;44(5):1322–53. doi: 10.1038/s44318-024-00358-0 (PMC11876699; doi:10.1038/s44318-024-00358-0)
Supplement: Supplementary file 1 — Appendix [file 44318_2024_358_MOESM1_ESM.pdf]

Appendix to

Elongator is a microtubule polymerase selective for polyglutamylated tubulin

Vicente J. Planelles-Herrero, Mariya Genova, Lara K. Krüger, Alice Bittleston, Kerrie E. McNally, Tomos E. Morgan, Gianluca Degliesposti, Maria M. Magiera, Carsten Janke and Emmanuel Derivery

Table of content:

Appendix Tables 2
Appendix Table S1 2
Appendix Table S2. 4
Appendix Figures 5
Appendix Figure S1 5
Appendix Figure S2 6
Appendix Figure S3 8
Appendix Figure S4 9
Appendix Figure S5 10
Appendix Figure S6 12
Appendix Figure S7 13

Appendix Tables

Appendix Table S1. Detected crosslinks between Elp123 and Elp456 from a His-PC-SNAP-Elongator prep from *Drosophila* S2 cells.

Protein1	Protein2	Position 1	Position 2	Score
dElp1	dElp1	168	230	53
dElp1	dElp1	896	870	139
dElp1	dElp1	966	915	92
dElp1	dElp1	1071	696	145
dElp1	dElp1	1184	930	82
dElp1	dElp1	302	377	136
dElp1	dElp1	696	740	62
dElp1	dElp1	870	896	151
dElp1	dElp1	915	966	126
dElp1	dElp1	930	1115	79
dElp1	dElp1	930	1161	124
dElp1	dElp1	930	1184	136
dElp1	dElp1	1115	1235	158
dElp1	His-PC-dElp3S	854	97	79
dElp1	dElp4	540	256	149
dElp1	dElp4	1115	364	64
dElp1	dElp6_anon-i1	631	228	187
dElp2	dElp2	97	58	102
dElp2	dElp2	97	756	134
dElp2	dElp2	601	456	127
dElp2	dElp2	756	58	113
dElp2	dElp2	58	97	103
His-PC-dElp3S	His-PC-dElp3S	69	106	72
His-PC-dElp3S	His-PC-dElp3S	75	61	172
His-PC-dElp3S	His-PC-dElp3S	97	60	170
His-PC-dElp3S	His-PC-dElp3S	97	106	59
His-PC-dElp3S	His-PC-dElp3S	102	60	92
His-PC-dElp3S	His-PC-dElp3S	61	75	126
His-PC-dElp3S	His-PC-dElp3S	69	106	92
His-PC-dElp3S	His-PC-dElp3S	69	419	75
His-PC-dElp3S	His-PC-dElp3S	106	360	63
dElp4	dElp4	169	256	75
dElp4	dElp4	276	11	100
dElp4	dElp5_poly	64	159	130
dElp4	dElp5_poly	364	159	126

dElp4	dElp5_poly	364	159	99
dElp4	His-PC-dElp3S	364	360	89
dElp4	His-PC-dElp3S	364	360	140
dElp4	dElp4	11	276	99
dElp5_poly	dElp5_poly	9	159	109
dElp5_poly	dElp5_poly	27	175	81
dElp5_poly	dElp5_poly	179	151	83
dElp5_poly	dElp5_poly	179	151	111
dElp5_poly	dElp6_anon-i1	27	161	132
dElp5_poly	His-PC-dElp3S	151	31	103
dElp5_poly	His-PC-dElp3S	151	61	76
dElp5_poly	His-PC-dElp3S	151	75	72
dElp6_anon-i1	dElp6_anon-i1	209	228	50
dElp6_anon-i1	dElp6_anon-i1	209	250	113
dElp6_anon-i1	His-PC-dElp3S	199	419	88
dElp6_anon-i1	His-PC-dElp3S	209	419	56
dElp6_anon-i1	His-PC-dElp3S	199	419	187
dElp6_anon-i1	His-PC-dElp3S	209	419	174

Appendix Table S2. Detected crosslinks between Elp456 and the Glu_{10} peptide.

	Protein A	Residue	Linked Residue	Glu10 peptide position	Highest Score
Crosslinker: SDA	Elp4	11	T	10	21.356
	Elp4	126	Y	10	17.912
	Elp4	126	Y	7	20.023
	Elp4	129	L	1	21.5
	Elp4	240	T	6	21.473
	Elp4	366	Y	9	18.09
	Elp4	366	Y	6	17.519
	Elp4	366	Y	7	16.736
	Elp4	366	Y	10	16.394

	Protein A	Residue	Linked Residue	Glu10 peptide position	Highest Score
Crosslinker: EDC	Elp4	6	K	4	15.412
	Elp4	11	K	4	13.886
	Elp4	11	K	5	19.585
	Elp4	11	K	5	14.296
	Elp4	11	K	7	28.804
	Elp4	11	K	7	19.046
	Elp4	399	K	7	15.785
	Elp4	399	K	7	13.279
	Elp4	399	G	7	13.176
	Elp4	399	K	7	13.105
	Elp4	399	K	7	12.925
	Elp4	399	K	7	23.405
	Elp5	159	K	8	21.721
	Elp5	159	K	8	21.02
	Elp5	159	K	8	20.195
	Elp5	179	K	8	18.347
	Elp5	179	K	9	18.138
	Elp5	179	K	9	16.537
	Elp5	194	K	9	16.196
	Elp6	217	K	9	12.918
	Elp6	221	K	10	21.75
	Elp6	221	K	10	17.116
	Elp6	240	K	10	16.251
	Elp6	240	Y	10	13.527
	Elp6	240	K	10	35.176

Appendix Figures

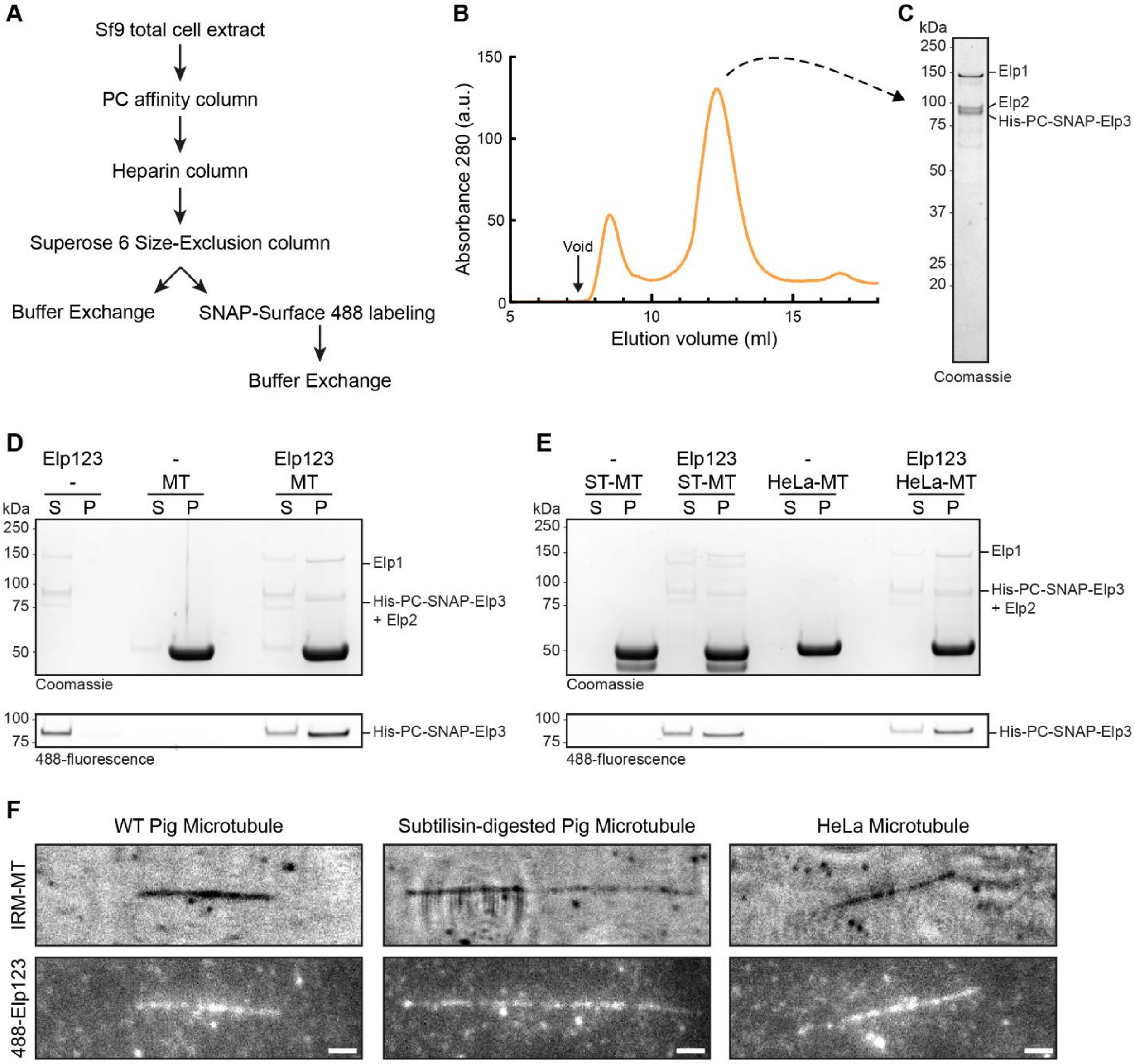

Appendix Figure S1. Elp123 purification and characterization

(A, B, C) Purification and fluorescent labelling of Elp123 from Sf9 cells (see methods). **(D, E)** Coomassie (top) and 488-fluorescence (bottom) analysis of *in vitro* co-sedimentation assays with microtubules stabilized with taxol (5 µM tubulin, 40 µM taxol) and 160 nM Elp123. In the presence of pig brain microtubules (D), partially subtilisin-digested pig brain microtubules (E) and HeLa S3 microtubules (E), all Elp123 is found in the pellet. P, pellet; S, supernatant. **(F)** 488-Elp123 decorates wild-type pig microtubules (left), partially subtilisin-digested pig brain microtubules (middle) and HeLa S3 microtubules (right). Scale bars = 2 µm.

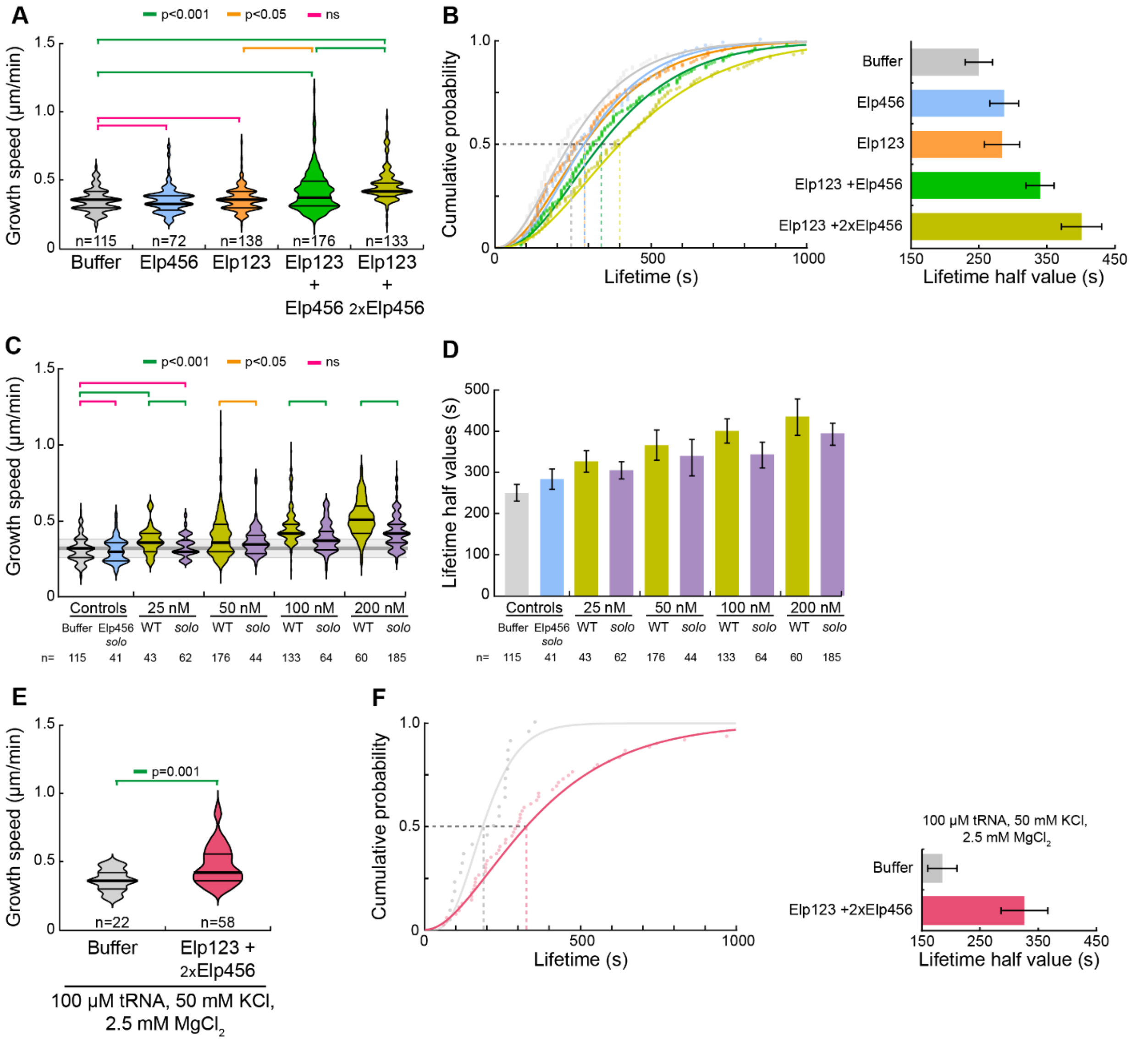

Appendix Figure S2. Elongator and sub-complexes effect on the microtubules minus-ends

(A, B) Effect of the indicated conditions on the growth speed (A) and lifetime (B) of microtubules at the minus end imaged by TIRFM in the presence of 16 μM GTP-tubulin (10% HiLyte 647 labelled). Elongator concentrations are: 100 nM Elp456 ("Elp456"), 50 nM $(His)_6$-PC-SNAP-Elp123 ("Elp123"), 50 nM Elp456+50 nM $(His)_6$-PC-SNAP-Elp123 ("Elp123+Elp456"), and 100 nM Elp456+50 nM $(His)_6$-PC-SNAP-Elp123 ("Elp123+2xElp456"). **(C, D)** Effect of the indicated proteins and concentrations on the growth speed (C) and lifetime (D) of microtubules at the minus end measured by TIRFM in the presence of 16 μM GTP-tubulin (10% HiLyte 647 labelled). Elongator concentrations are: 100 nM Elp456 *solo* ("Elp456 solo"), 100 nM Elp456 *solo*+50 nM $(His)_6$-PC-SNAP-Elp123 ("Elp123+Elp456 solo"), and 100 nM Elp456 wild-type+50 nM $(His)_6$-PC-SNAP-Elp123 ("Elp123+Elp456").. **(E, F)** Effect of the indicated conditions on the growth speed (E) and lifetime (F) of microtubules at the minus end imaged by TIRFM in the presence of "Control" and "Elp123 + 2x(Elp456)" datasets are the same as the ones presented in Fig. 4B-C, shown here for convenience. In high tRNA buffer (100 μM tRNA, 2.5 mM MgATP), Elongator effect on microtubules is indistinguishable from BRB80 buffer with no tRNA (Fig. 4B, C) for both microtubule growth (G) and lifetime

(H). P values for a Kruskal-Wallis test followed by Dunn's multiple comparison test are indicated. Dashed line represents an increase of ~1.4 in the speed of microtubule growth[30]. Thick line, median; thin line, quartile. Microtubule lifetime estimate ± error from the bootstrapped mean lifetimes (see methods) are indicated in the right panel. n, number of microtubule-growing events analysed.

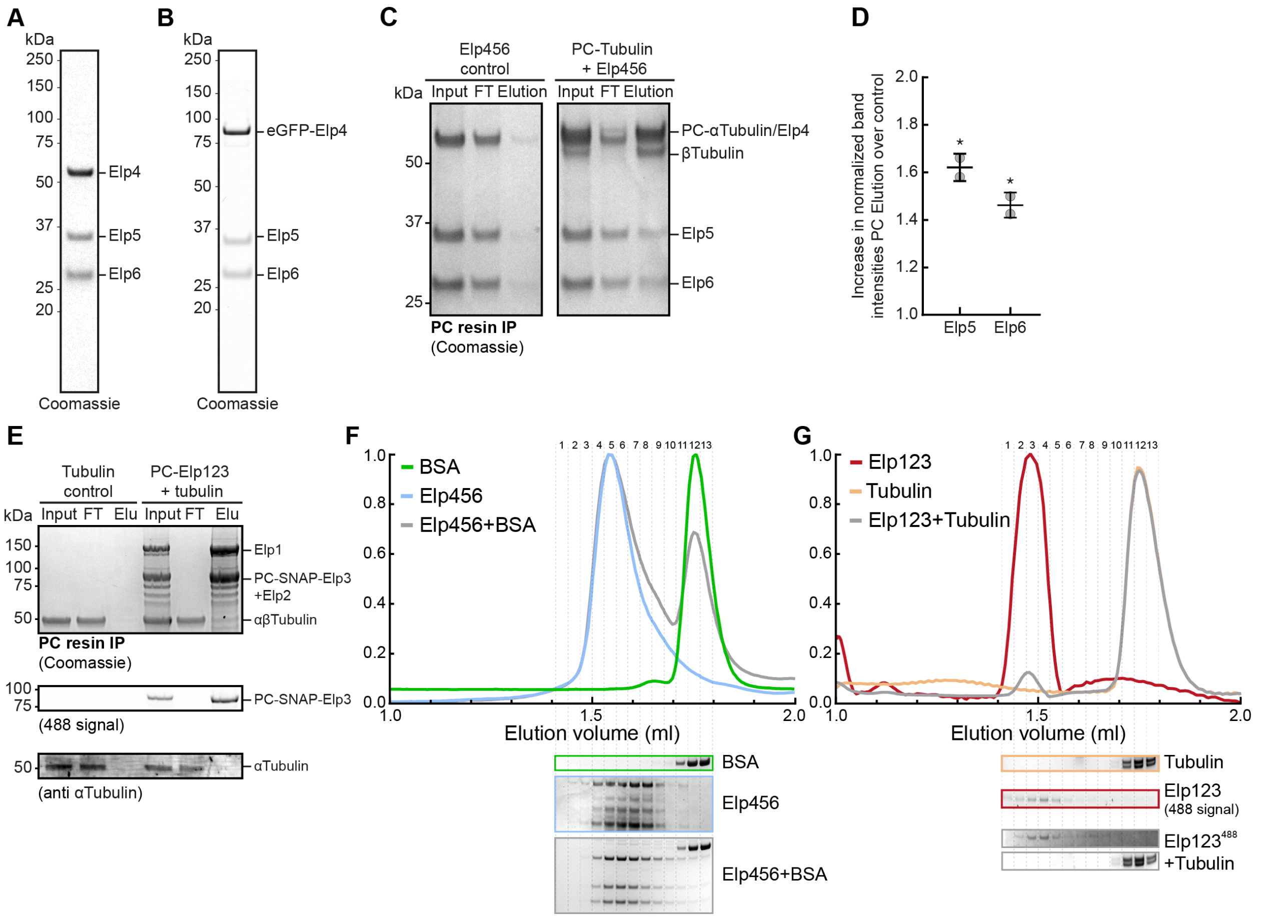

Appendix Figure S3. Elongator sub-complexes binding to tubulin

(A, B) Purification of Elp456 and eGFP-Elp456 from *E. coli* cells. **(C)** Elp456 binds to recombinant *Drosophila* PC-tagged α1β1-tubulin heterodimers. Immunoprecipitation assay using a resin coated with anti-PC antibodies. Elp456 is detected in the elution only in the presence of PC-tubulin. Protein presence was analysed using coomassie blue. **(D)** Quantification of the band intensities in (C). For every independent experiment (n=2), the band intensities in the "Elution" fraction were normalized against the input, and the significance for a t-test between experiment and control is indicated with a * (p-values are 0.028 and 0.045 for Elp5 and Elp6, respectively). Displayed is the increase in intensity for Elp5 and Elp6 in the experiment over the control. **(E)** Elp123 does not bind to tubulin. Pig brain tubulin cannot be detected eluting from PC-resin in the absence or presence of PC-Elp123. A very high concentration of Elp123 was used to favour any possible weak binding between tubulin and Elp123. The coomassie blue analysis was complemented with fluorescence (488-, labelling Elp123) and western blot analysis using antibodies against αtubulin. **(F)** Elp456 does not bind to BSA in the same conditions as in Fig 3B. Note that the apparent elution volume for Elp456 is different due to the use of a different setup. The normalized elution volume for Elp456 (defined as $(V_{elution}-V_{void})/V_{void}$) is 0.74 in Fig 3B and 0.75 in Appendix Figure S3F. **(G)** Elp123 does not bind to α1β1-tubulin heterodimers in the same conditions as in Fig 3B.

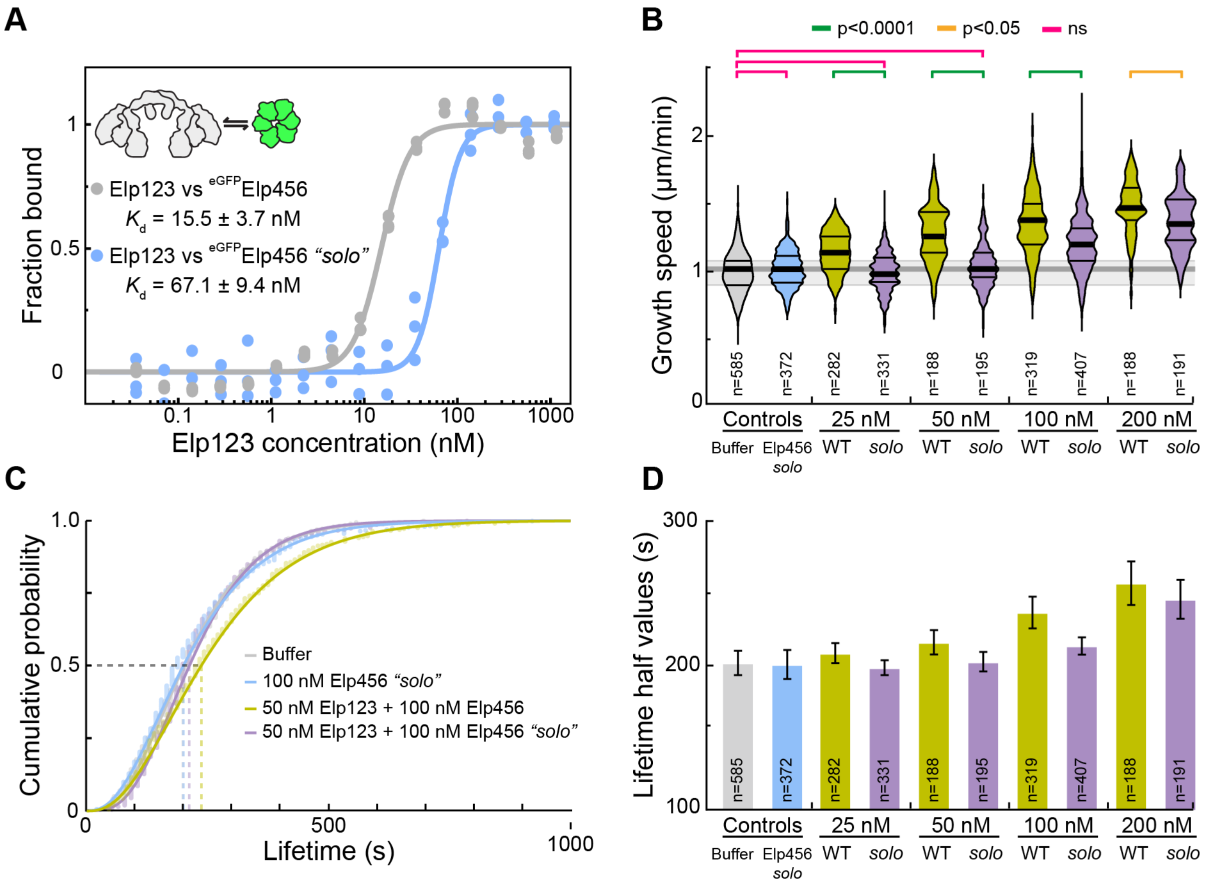

Appendix Figure S4. Elongator sub-complexes binding to tubulin

(A) Measurement of the Elp123 and eGFP-Elp456 WT and *solo* mutant interaction using microscale thermophoresis (see methods). Calculated dissociation constant (K_d) values are indicated (mean ± s.d.; n=3). The calculated K_d for the Elp456 *solo* mutant is 4.3 times lower than for the wild-type. **(B, C, D)** Effect of the indicated proteins and concentrations on the growth speed (C) and lifetime (D, E) of microtubules at the plus end measured by TIRFM in the presence of 16 µM GTP-tubulin (10% HiLyte 647 labelled). Elongator concentrations are: 100 nM Elp456 *solo* ("Elp456 solo"), 100 nM Elp456 *solo*+50 nM $(His)_6$-PC-SNAP-Elp123 ("Elp123+Elp456 solo"), and 100 nM Elp456 wild-type+50 nM $(His)_6$-PC-SNAP-Elp123 ("Elp123+Elp456"). n, number of microtubule-growing events analysed. Similar results were observed at the minus end (see text and Sup. Fig. S3C, D). **(B)** P values for a Kruskal-Wallis test followed by Dunn's multiple comparison test are indicated. Dashed line represents an increase of ~1.4 in the speed of microtubule growth[31]. Thick line, median; thin line, quartile. **(D)** Microtubule lifetime estimate ± error from the bootstrapped mean lifetimes (see methods).

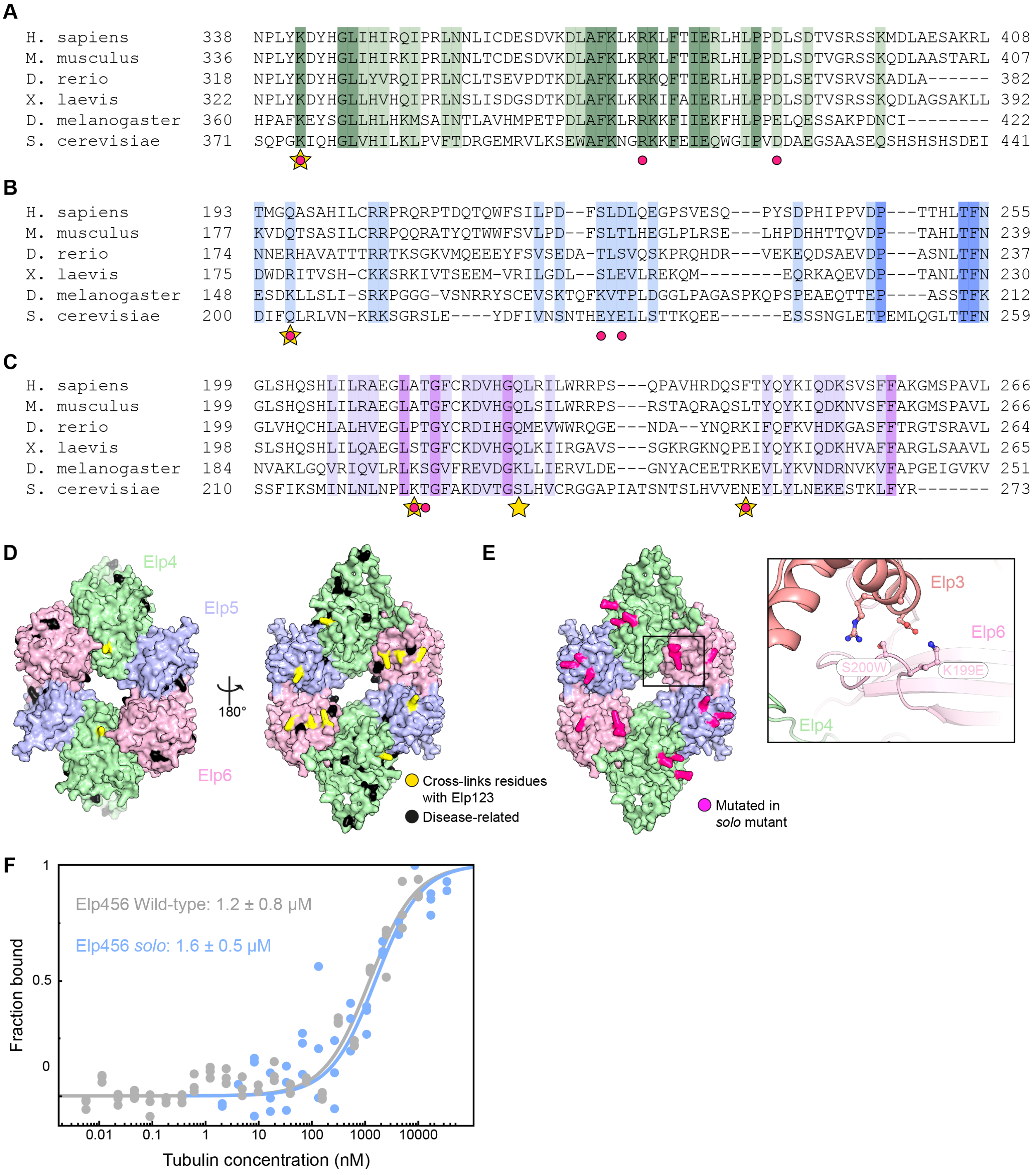

Appendix Figure S5. Elongator-tubulin complex reconstitution on microtubules

(A, B, C) Protein sequence alignments of regions of Elp4 (A), Elp5 (B) and Elp6 (C) surrounding detected crosslinks between Elp123 and Elp456 (E) (see also methods). Dark colours: residue strictly conserved. Lighter colours: partially conserved. Residue number and species used for the alignments are indicated. Yellow stars highlight detected crosslinks. Magenta dots indicate residues mutated in Elp456 *solo* mutant: Elp4 K364E, R397E, E410R; Elp5 K151E, K179E, T181A; Elp6 K119E, S200W, K228E. **(D, E)** Alphafold model

of *Drosophila melanogaster* Elp456 (see methods). **(D)** Detected crosslinks with Elp123 are highlighted in yellow. Disease-related residues found in ActiveDriverDB database. **(E)** Mutated residues in Elp456 *solo* mutant. Inset shows the interface between Elp3 and Elp6 from ref[1], highlighting how the mutations K199E and S200W introduced in Elp456 *solo* might perturb this interaction. **(F)** Elp456 *solo binds* to recombinant *Drosophila* α1β1-tubulin heterodimers with wild-type affinity. Calculated dissociation constant (K_d) values are indicated (mean ± s.d.; n=3).

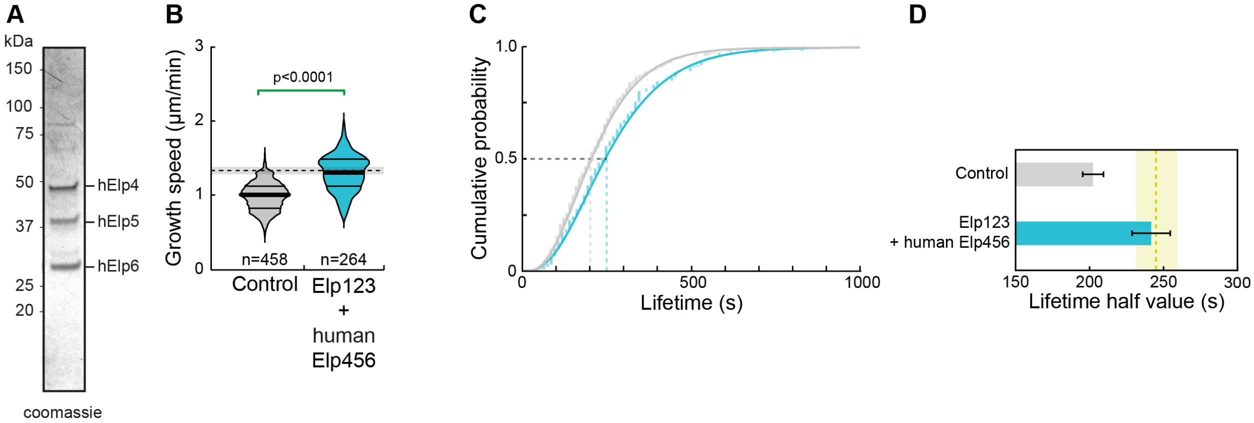

Appendix Figure S6. Elongator effect on microtubules is not *Drosophila* specific

(A) Purification of human Elp456. **(B, C, D)** Effect of the human Elp456, together with *Drosophila* Elp123 on the growth speed (B) and lifetime (C, D) of microtubules at the plus end. n, number of microtubule-growing events analysed. **(B)** P values for a Kruskal-Wallis test followed by Dunn's multiple comparison test are indicated. Dashed line represents an increase of ~1.4 in the speed of microtubule growth[2]. Thick line, median; thin line, quartile. **(C, D)** Lifetime estimate ± error from the bootstrapped mean lifetimes (see methods) are indicated in the right panel. Green shaded region indicates the values obtained with wild-type Elp456 (Fig 4C). Dashed line represents the median, and the shaded region the quartiles.

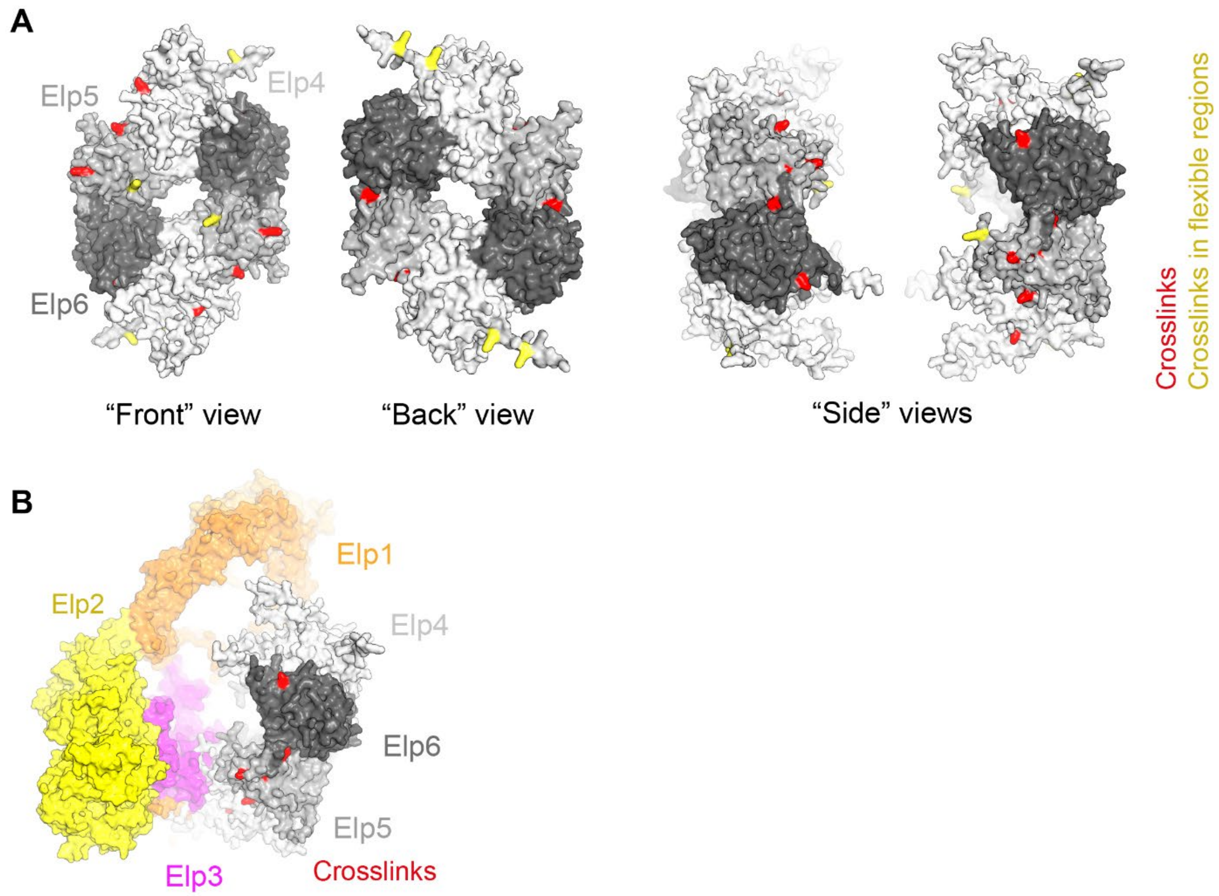

Appendix Figure S7. Characterization of the tubulin-Elp456 binding
(A) Alphafold model of *Drosophila melanogaster* Elp456 (see methods). Detected crosslinks with a linear 10 Glu peptide (Glu_{10}) highlighted in red (in the rigid core of the Elp456 surface) and yellow (in flexible regions). **(B)** Relative location of the cross-linked residues in the full assembled Elongator complex (using PDB 8ASV as a reference).

References for supplementary information

1. Jaciuk, M. *et al.* Cryo-EM structure of the fully assembled Elongator complex. *Nucleic Acids Res* **1**, (2023).

2. Planelles-Herrero, V. J. *et al.* Elongator stabilizes microtubules to control central spindle asymmetry and polarized trafficking of cell fate determinants. *Nat Cell Biol* **24**, 1606–1616 (2022).
