# Supplementary figures and images for: Elongator is a microtubule polymerase selective for polyglutamylated tubulin (part 1 of 2)
Source: EMBO J. 2025 Jan 15;44(5):1322–53. doi: 10.1038/s44318-024-00358-0 (PMC11876699; doi:10.1038/s44318-024-00358-0)

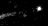

Supplement: Supplementary file 3 — Source data Fig. 1 [file 44318_2024_358_MOESM3_ESM.zip › Figure 1/1C/Elongator.tif]

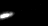

Supplement: Supplementary file 3 — Source data Fig. 1 [file 44318_2024_358_MOESM3_ESM.zip › Figure 1/1C/Microtubule.tif]

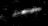

Supplement: Supplementary file 3 — Source data Fig. 1 [file 44318_2024_358_MOESM3_ESM.zip › Figure 1/1C/Tubulin.tif]

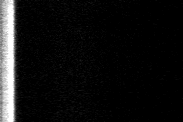

Supplement: Supplementary file 3 — Source data Fig. 1 [file 44318_2024_358_MOESM3_ESM.zip › Figure 1/1D/C1-kymograph.tif]

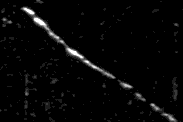

Supplement: Supplementary file 3 — Source data Fig. 1 [file 44318_2024_358_MOESM3_ESM.zip › Figure 1/1D/C2-kymograph-1.tif]

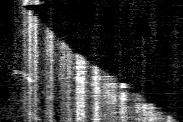

Supplement: Supplementary file 3 — Source data Fig. 1 [file 44318_2024_358_MOESM3_ESM.zip › Figure 1/1D/C3-kymograph-1.tif]

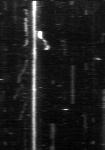

Supplement: Supplementary file 3 — Source data Fig. 1 [file 44318_2024_358_MOESM3_ESM.zip › Figure 1/1E/C1-1E.tif]

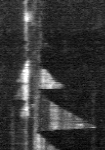

Supplement: Supplementary file 3 — Source data Fig. 1 [file 44318_2024_358_MOESM3_ESM.zip › Figure 1/1E/C2-1E.tif]

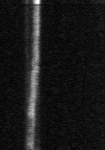

Supplement: Supplementary file 3 — Source data Fig. 1 [file 44318_2024_358_MOESM3_ESM.zip › Figure 1/1E/C3-1E.tif]

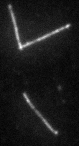

Supplement: Supplementary file 3 — Source data Fig. 1 [file 44318_2024_358_MOESM3_ESM.zip › Figure 1/1F/Composite.tif]

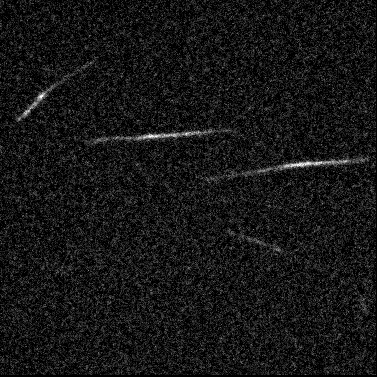

Supplement: Supplementary file 4 — Source data Fig. 2 [file 44318_2024_358_MOESM4_ESM.zip › Figure 2/A/C1-Composite.tif]

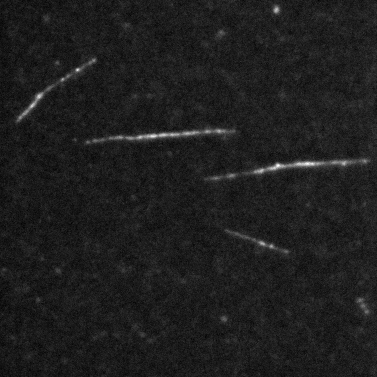

Supplement: Supplementary file 4 — Source data Fig. 2 [file 44318_2024_358_MOESM4_ESM.zip › Figure 2/A/C2-Composite.tif]

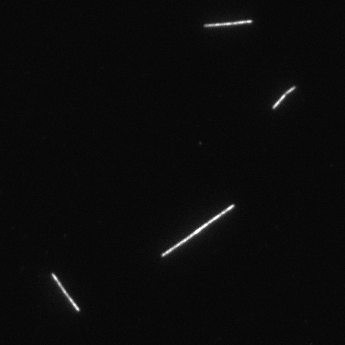

Supplement: Supplementary file 5 — Source data Fig. 3 [file 44318_2024_358_MOESM5_ESM.zip › Figure 3/A/C1-Lower panel.tif]

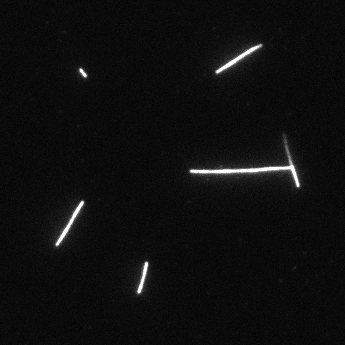

Supplement: Supplementary file 5 — Source data Fig. 3 [file 44318_2024_358_MOESM5_ESM.zip › Figure 3/A/C1-Upper panel.tif]

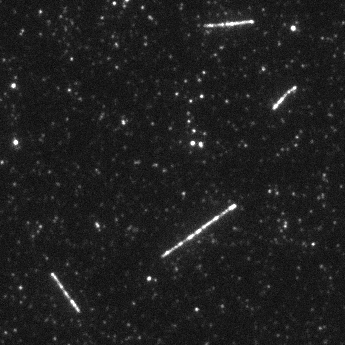

Supplement: Supplementary file 5 — Source data Fig. 3 [file 44318_2024_358_MOESM5_ESM.zip › Figure 3/A/C2-Lower panel.tif]

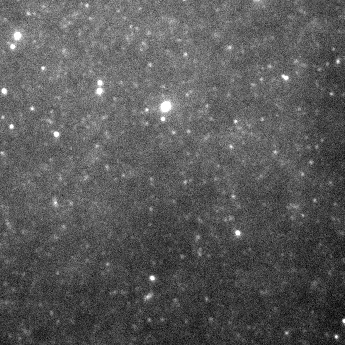

Supplement: Supplementary file 5 — Source data Fig. 3 [file 44318_2024_358_MOESM5_ESM.zip › Figure 3/A/C2-Upper panel.tif]

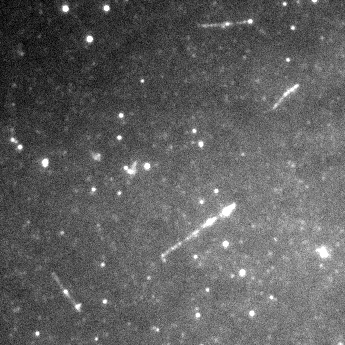

Supplement: Supplementary file 5 — Source data Fig. 3 [file 44318_2024_358_MOESM5_ESM.zip › Figure 3/A/C3-Lower panel.tif]

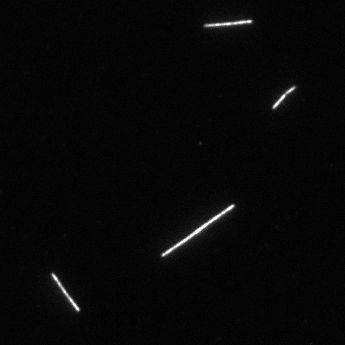

Supplement: Supplementary file 5 — Source data Fig. 3 [file 44318_2024_358_MOESM5_ESM.zip › Figure 3/A/Lower panel.tif]

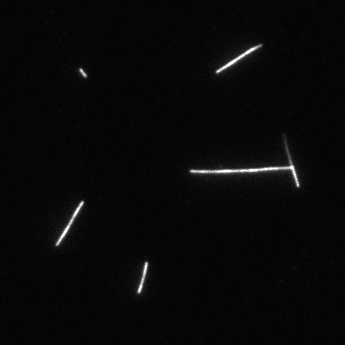

Supplement: Supplementary file 5 — Source data Fig. 3 [file 44318_2024_358_MOESM5_ESM.zip › Figure 3/A/Upper panel.tif]

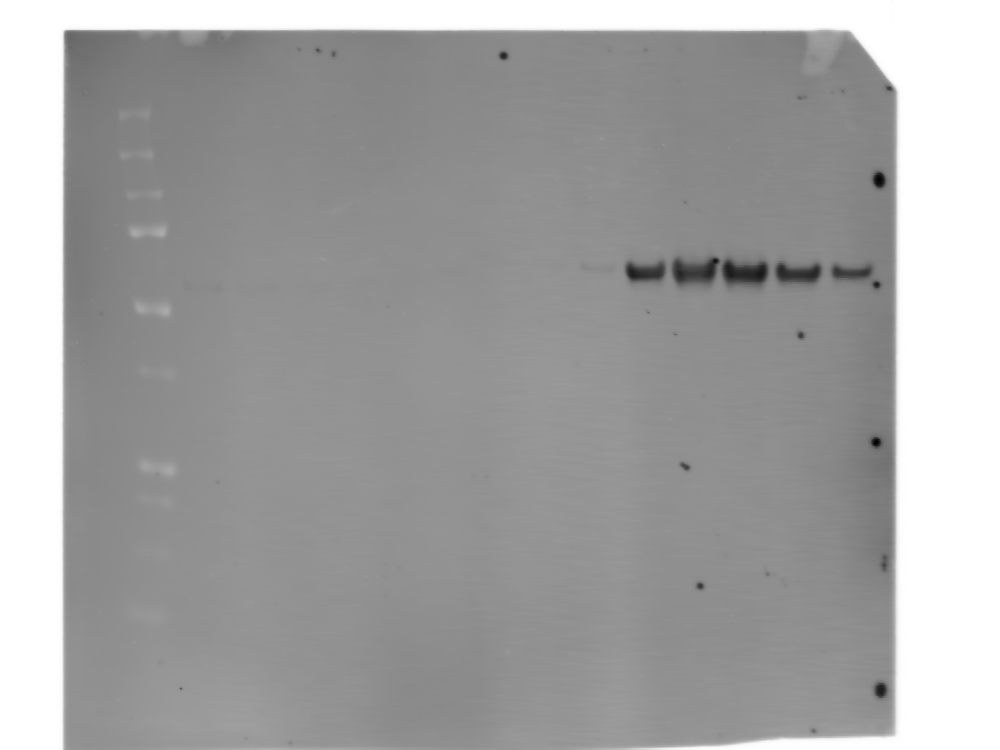

Supplement: Supplementary file 5 — Source data Fig. 3 [file 44318_2024_358_MOESM5_ESM.zip › Figure 3/C/aTubulin control (orange).tif]

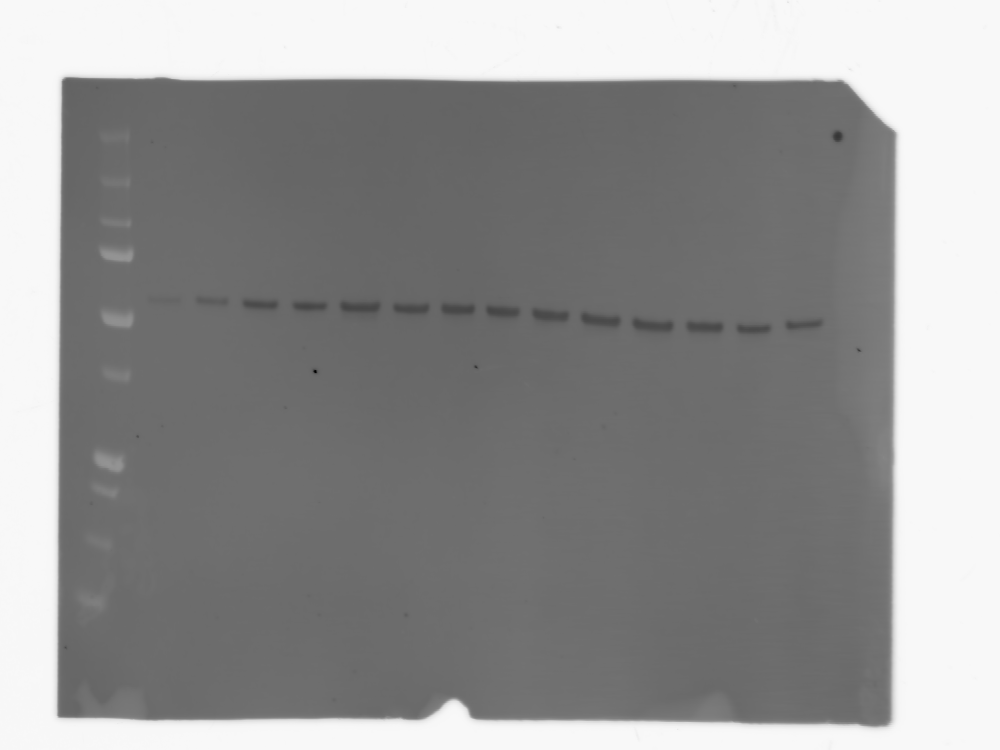

Supplement: Supplementary file 5 — Source data Fig. 3 [file 44318_2024_358_MOESM5_ESM.zip › Figure 3/C/aTubulin mix (black).tif]

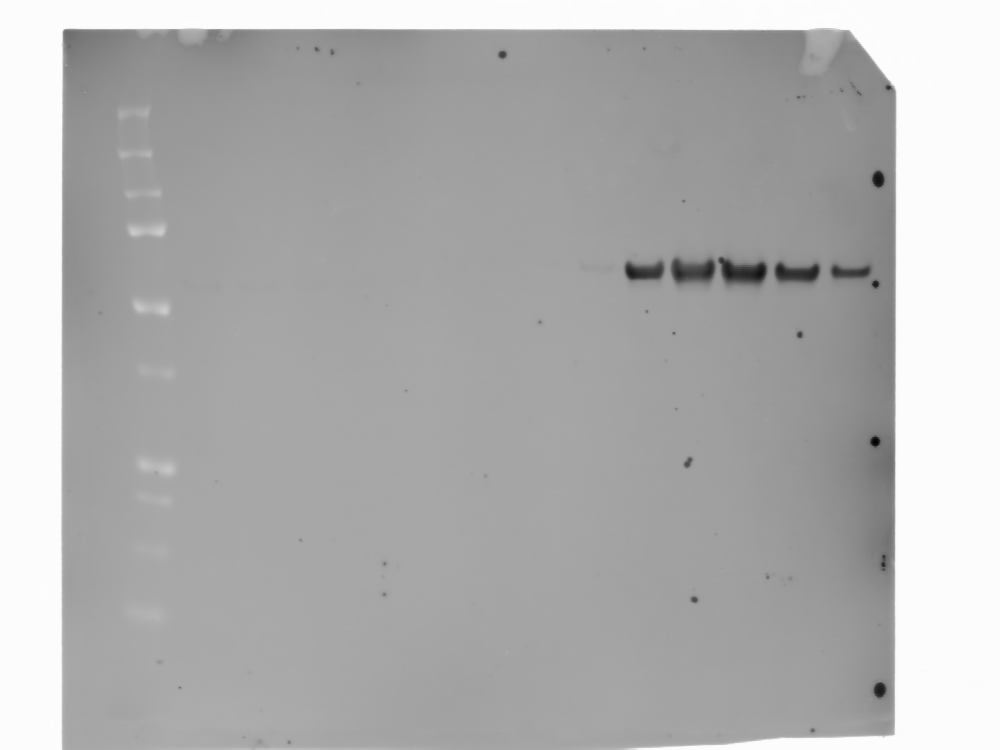

Supplement: Supplementary file 5 — Source data Fig. 3 [file 44318_2024_358_MOESM5_ESM.zip › Figure 3/C/bTubulin control (orange).tif]

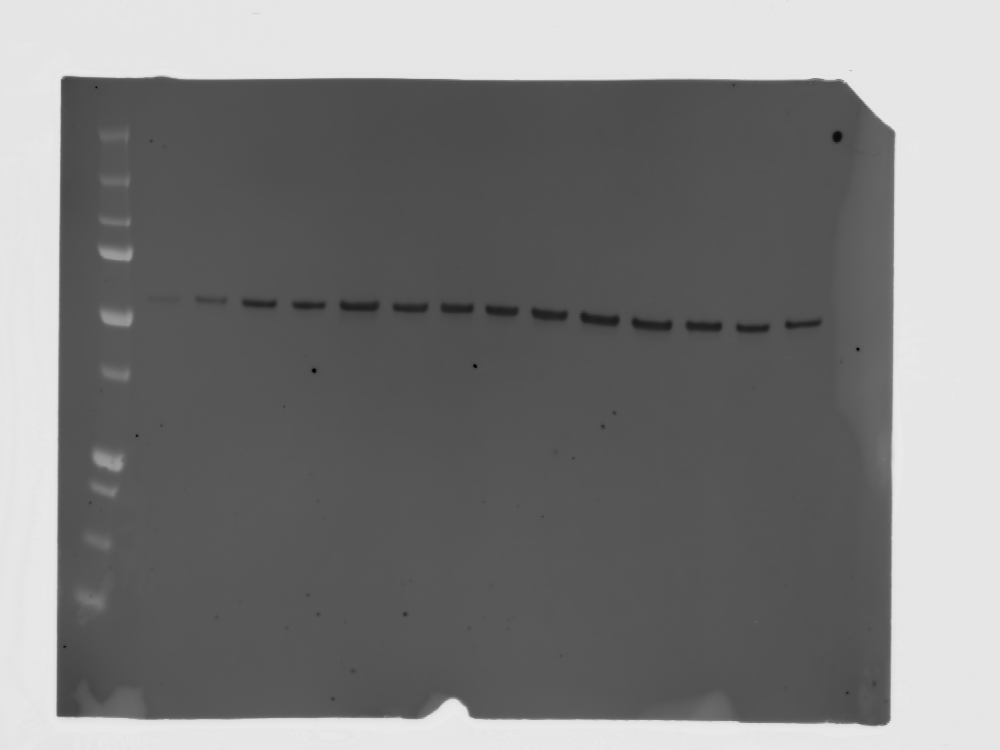

Supplement: Supplementary file 5 — Source data Fig. 3 [file 44318_2024_358_MOESM5_ESM.zip › Figure 3/C/bTubulin mix (black).tif]

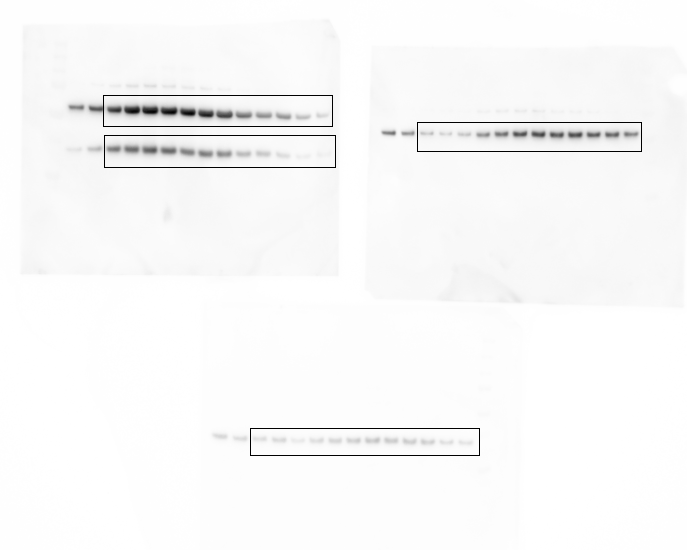

Supplement: Supplementary file 5 — Source data Fig. 3 [file 44318_2024_358_MOESM5_ESM.zip › Figure 3/C/Elp4 and Elp5 blots.tif]

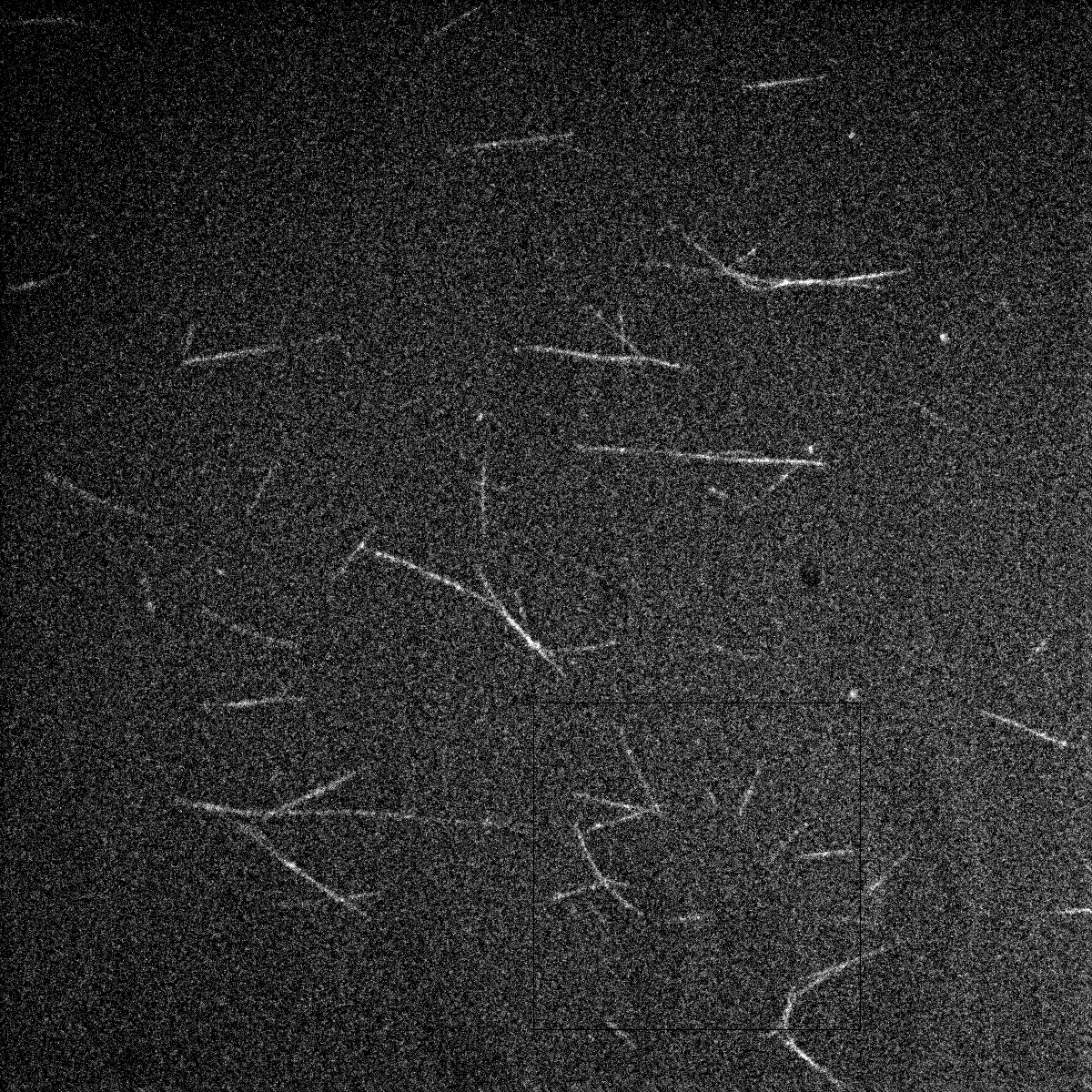

Supplement: Supplementary file 6 — Source data Fig. 4 [file 44318_2024_358_MOESM6_ESM.zip › Figure 4/A/C1-A.tif]

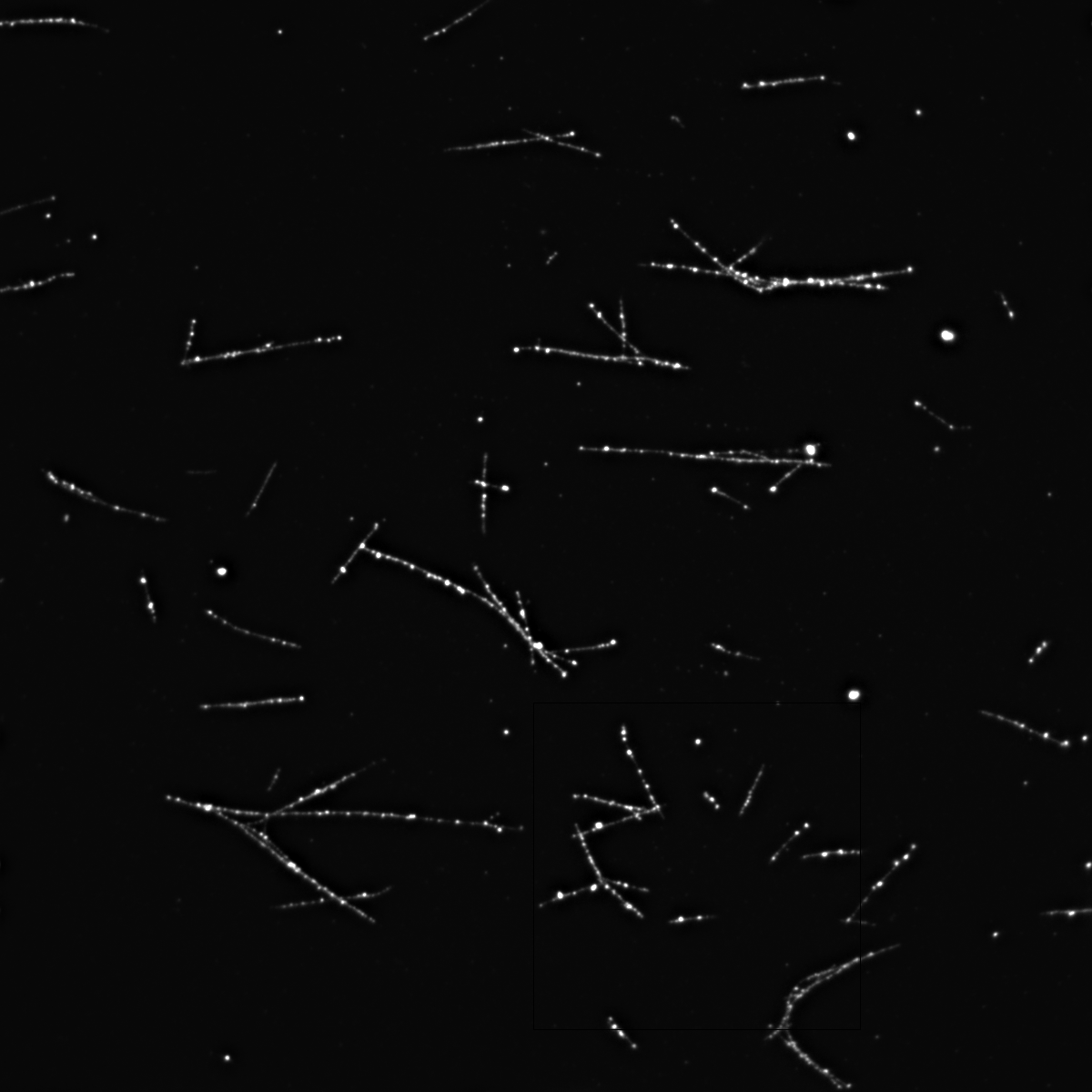

Supplement: Supplementary file 6 — Source data Fig. 4 [file 44318_2024_358_MOESM6_ESM.zip › Figure 4/A/C2-A.tif]

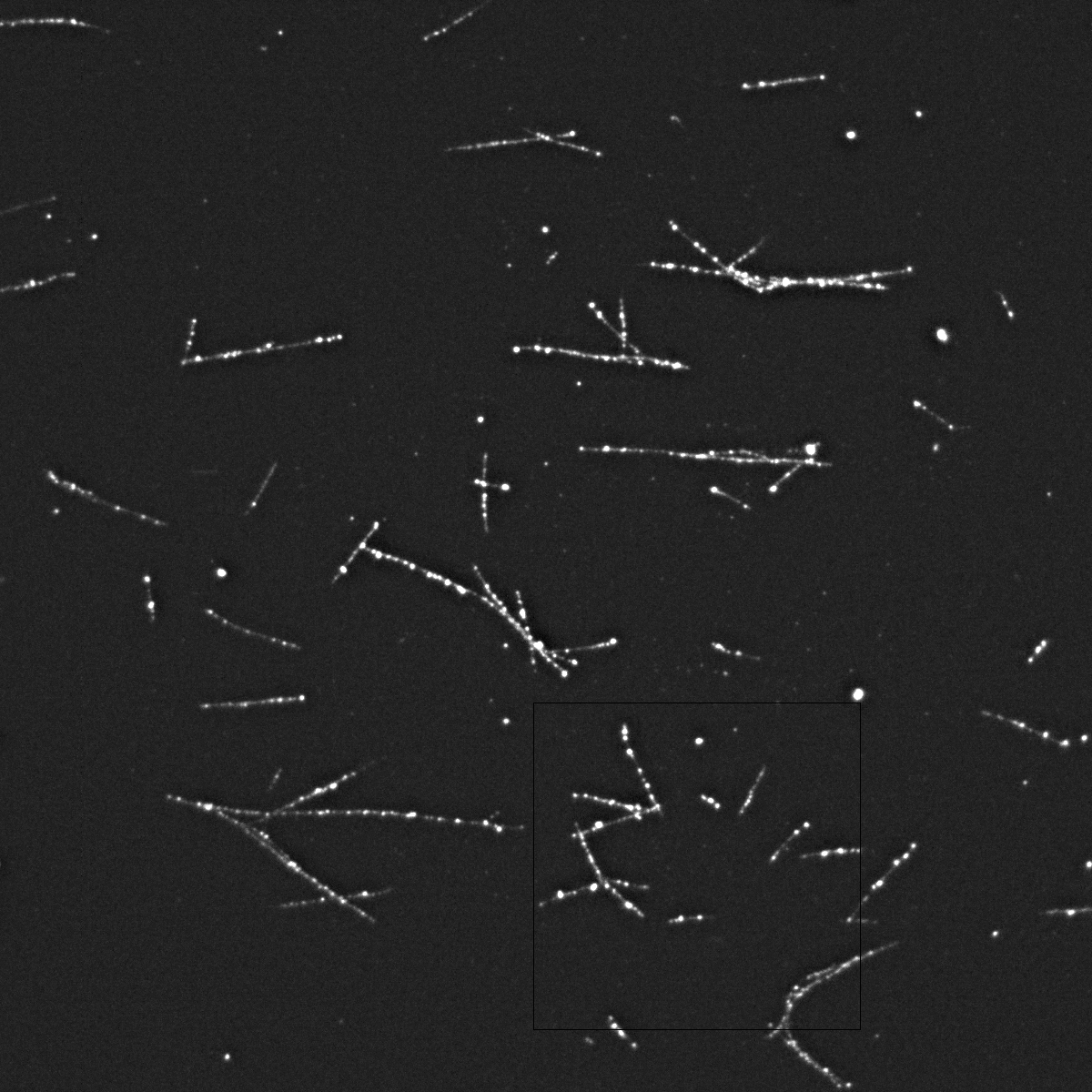

Supplement: Supplementary file 6 — Source data Fig. 4 [file 44318_2024_358_MOESM6_ESM.zip › Figure 4/A/C3-A.tif]

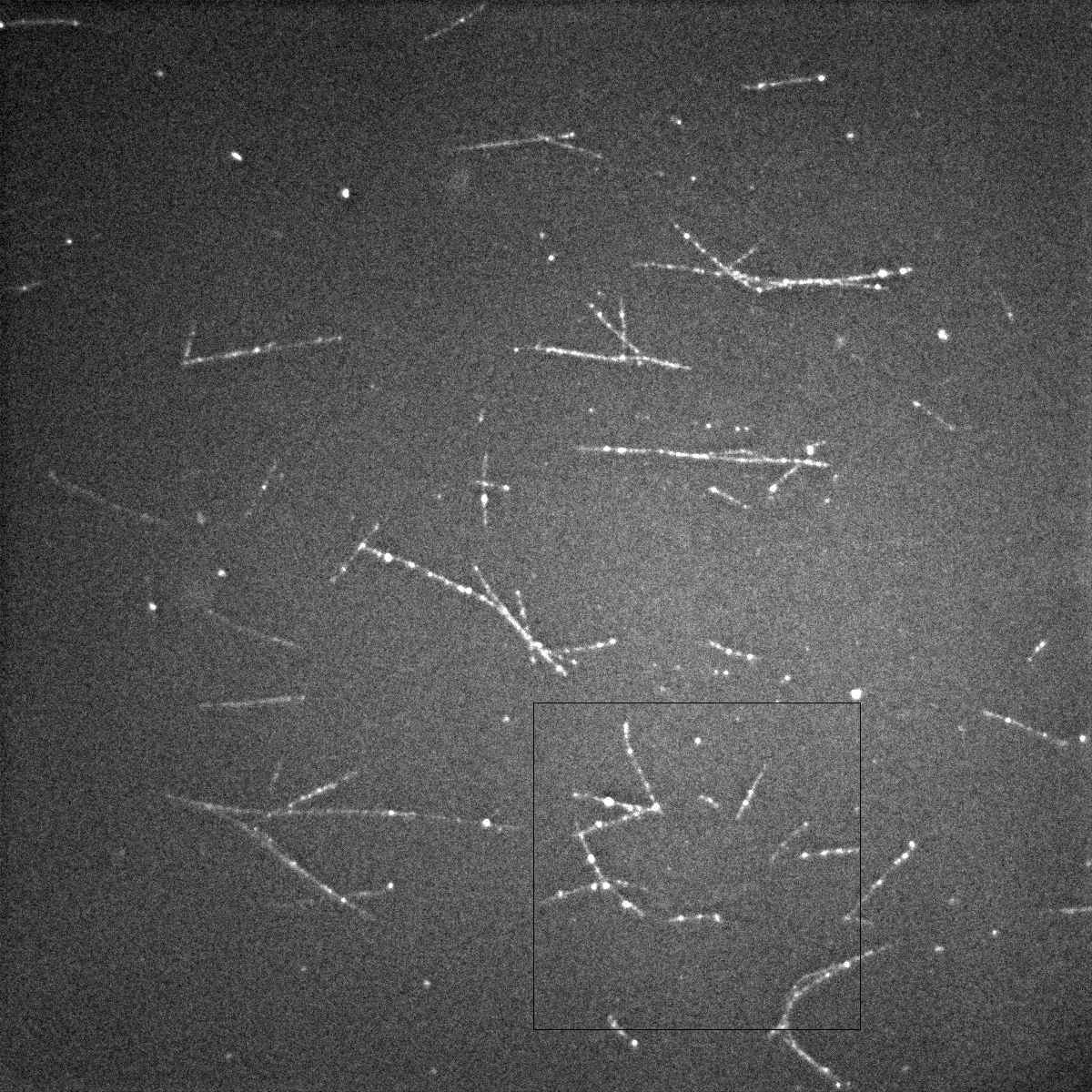

Supplement: Supplementary file 6 — Source data Fig. 4 [file 44318_2024_358_MOESM6_ESM.zip › Figure 4/A/C4-A.tif]

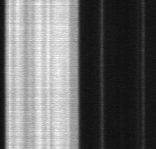

Supplement: Supplementary file 6 — Source data Fig. 4 [file 44318_2024_358_MOESM6_ESM.zip › Figure 4/D/C1-Buffer-1.tif]

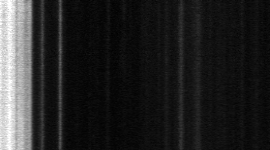

Supplement: Supplementary file 6 — Source data Fig. 4 [file 44318_2024_358_MOESM6_ESM.zip › Figure 4/D/C1-Elp123+2xElp456-1.tif]

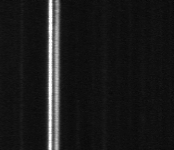

Supplement: Supplementary file 6 — Source data Fig. 4 [file 44318_2024_358_MOESM6_ESM.zip › Figure 4/D/C1-Elp123+Elp456-1.tif]

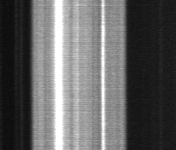

Supplement: Supplementary file 6 — Source data Fig. 4 [file 44318_2024_358_MOESM6_ESM.zip › Figure 4/D/C1-Elp123-1.tif]

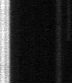

Supplement: Supplementary file 6 — Source data Fig. 4 [file 44318_2024_358_MOESM6_ESM.zip › Figure 4/D/C1-Elp456-1.tif]

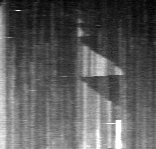

Supplement: Supplementary file 6 — Source data Fig. 4 [file 44318_2024_358_MOESM6_ESM.zip › Figure 4/D/C2-Buffer-1.tif]

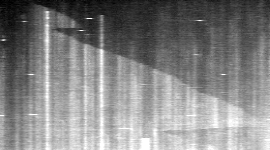

Supplement: Supplementary file 6 — Source data Fig. 4 [file 44318_2024_358_MOESM6_ESM.zip › Figure 4/D/C2-Elp123+2xElp456-1.tif]

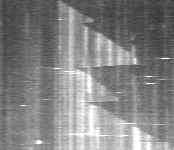

Supplement: Supplementary file 6 — Source data Fig. 4 [file 44318_2024_358_MOESM6_ESM.zip › Figure 4/D/C2-Elp123+Elp456-1.tif]

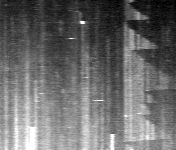

Supplement: Supplementary file 6 — Source data Fig. 4 [file 44318_2024_358_MOESM6_ESM.zip › Figure 4/D/C2-Elp123-1.tif]

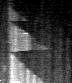

Supplement: Supplementary file 6 — Source data Fig. 4 [file 44318_2024_358_MOESM6_ESM.zip › Figure 4/D/C2-Elp456-1.tif]

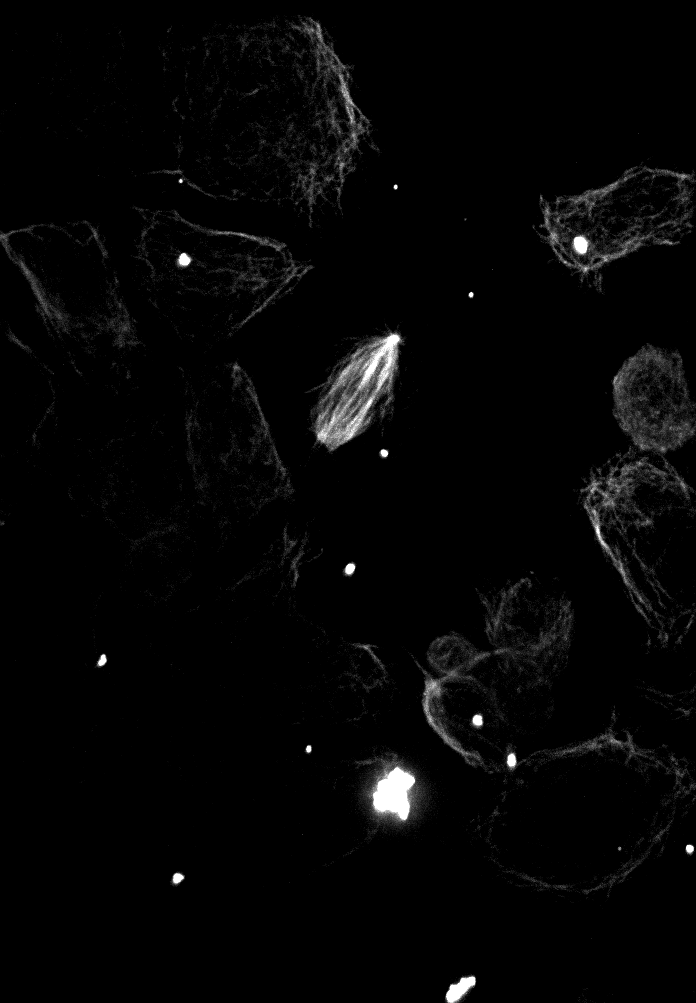

Supplement: Supplementary file 7 — Source data Fig. 5 [file 44318_2024_358_MOESM7_ESM.zip › Figure 5/E/C1-Anaphase A-projection-1.tif]

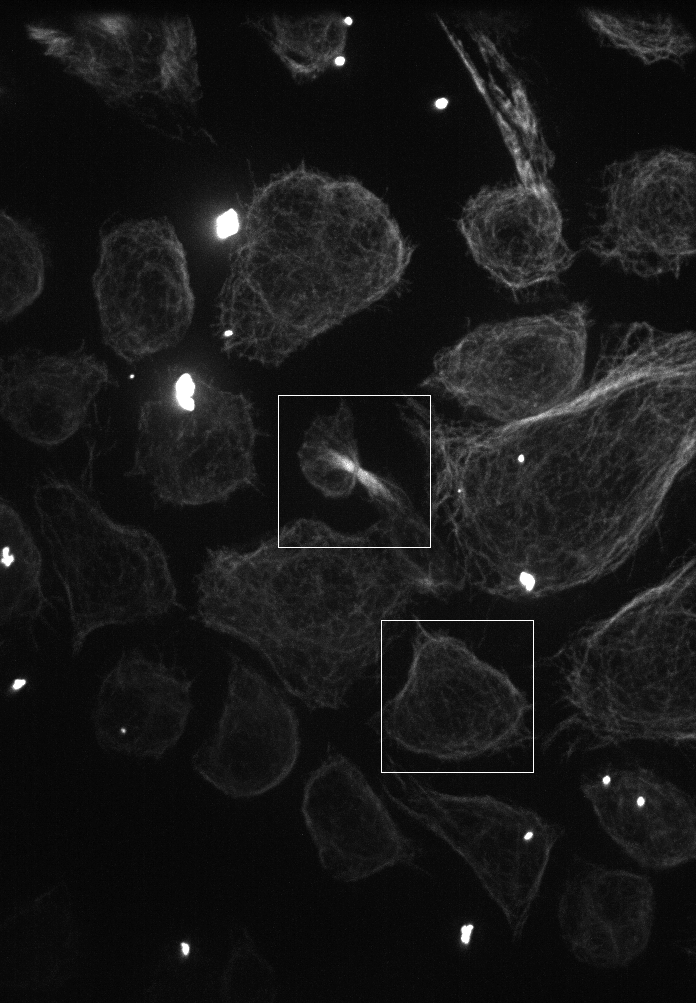

Supplement: Supplementary file 7 — Source data Fig. 5 [file 44318_2024_358_MOESM7_ESM.zip › Figure 5/E/C1-Interfase and Late anaphase-projection-1.tif]

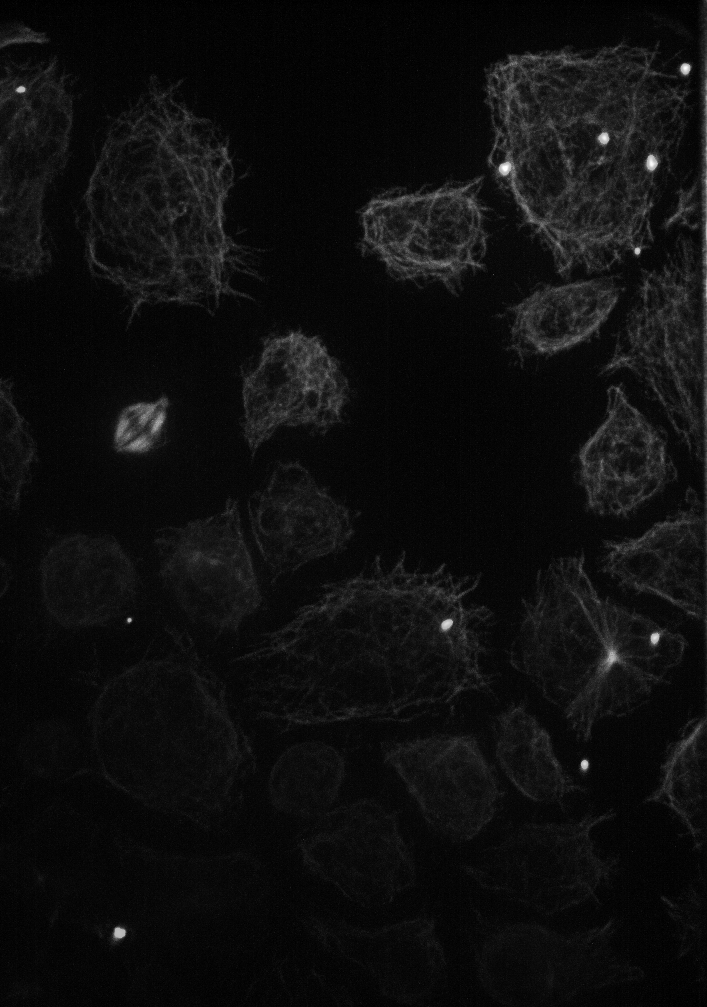

Supplement: Supplementary file 7 — Source data Fig. 5 [file 44318_2024_358_MOESM7_ESM.zip › Figure 5/E/C1-Metaphase-projection.tif]

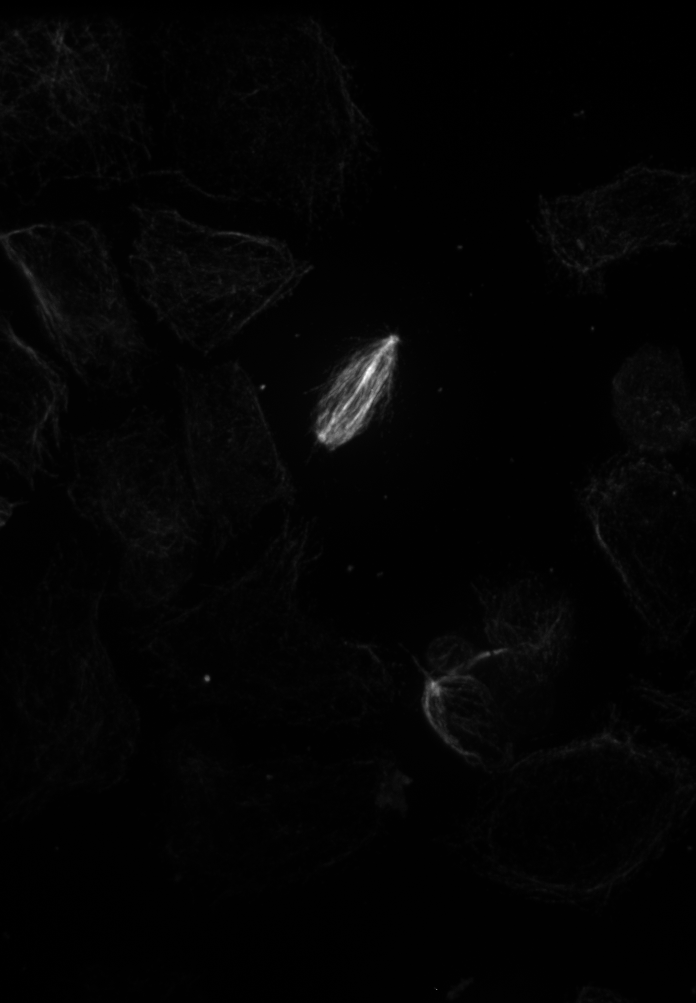

Supplement: Supplementary file 7 — Source data Fig. 5 [file 44318_2024_358_MOESM7_ESM.zip › Figure 5/E/C2-Anaphase A-projection-1.tif]

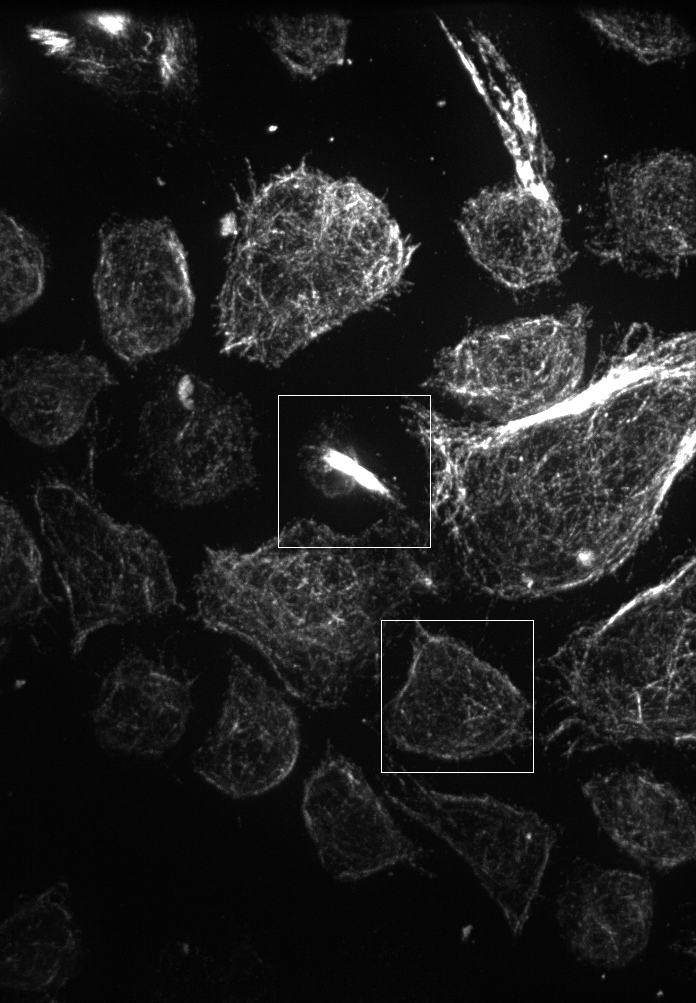

Supplement: Supplementary file 7 — Source data Fig. 5 [file 44318_2024_358_MOESM7_ESM.zip › Figure 5/E/C2-Interfase and Late anaphase-projection-1.tif]

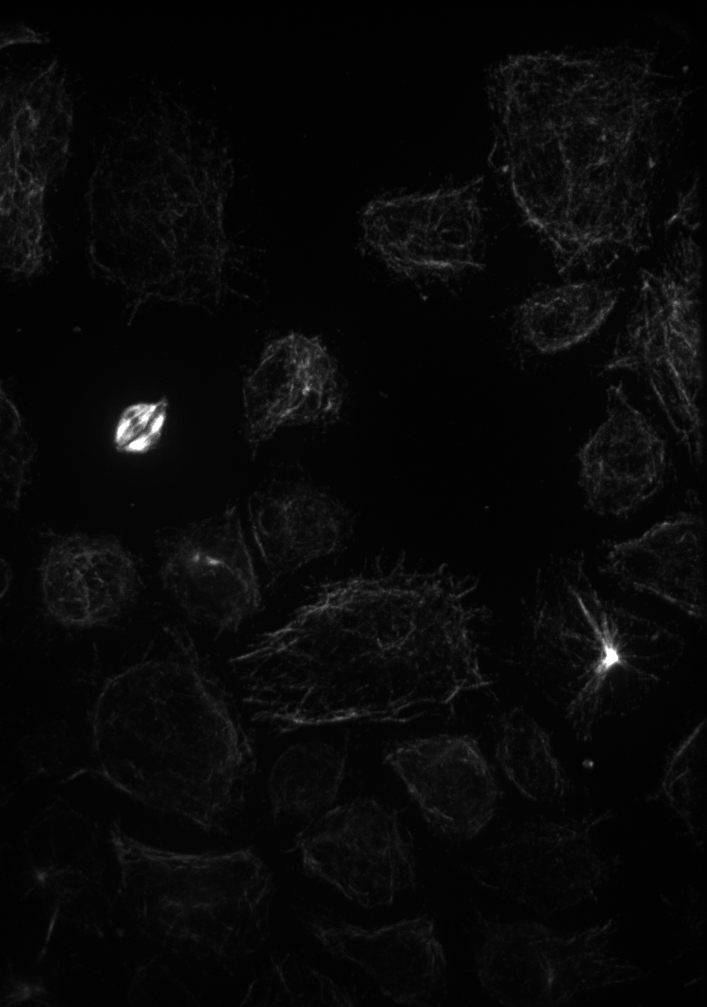

Supplement: Supplementary file 7 — Source data Fig. 5 [file 44318_2024_358_MOESM7_ESM.zip › Figure 5/E/C2-Metaphase-projection-1.tif]

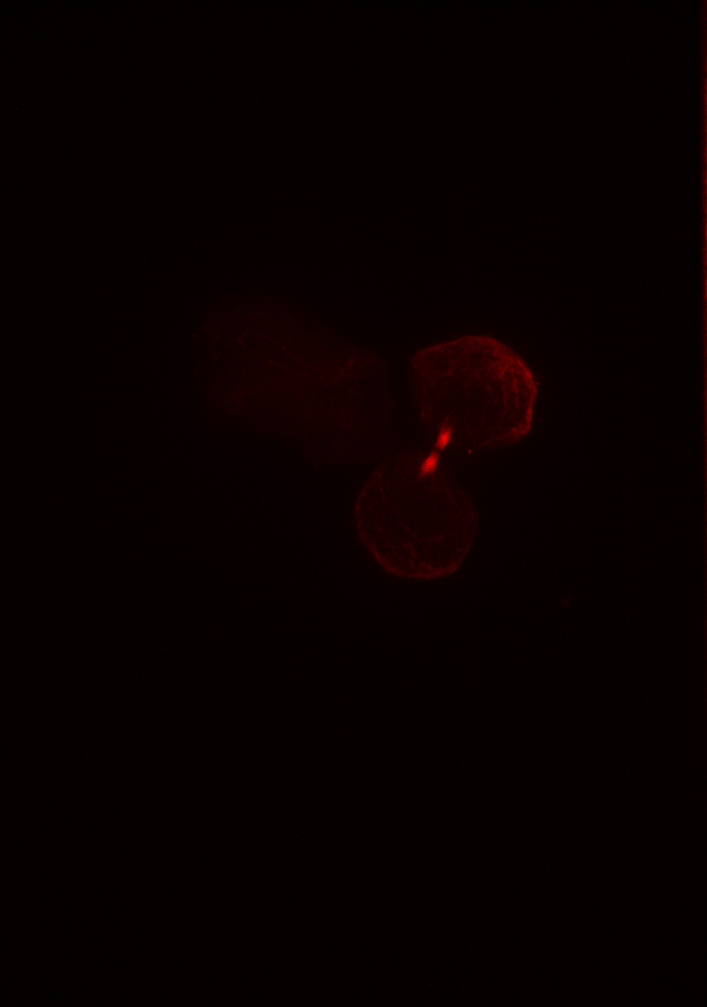

Supplement: Supplementary file 7 — Source data Fig. 5 [file 44318_2024_358_MOESM7_ESM.zip › Figure 5/F/C1-Anaphase-1.tif]

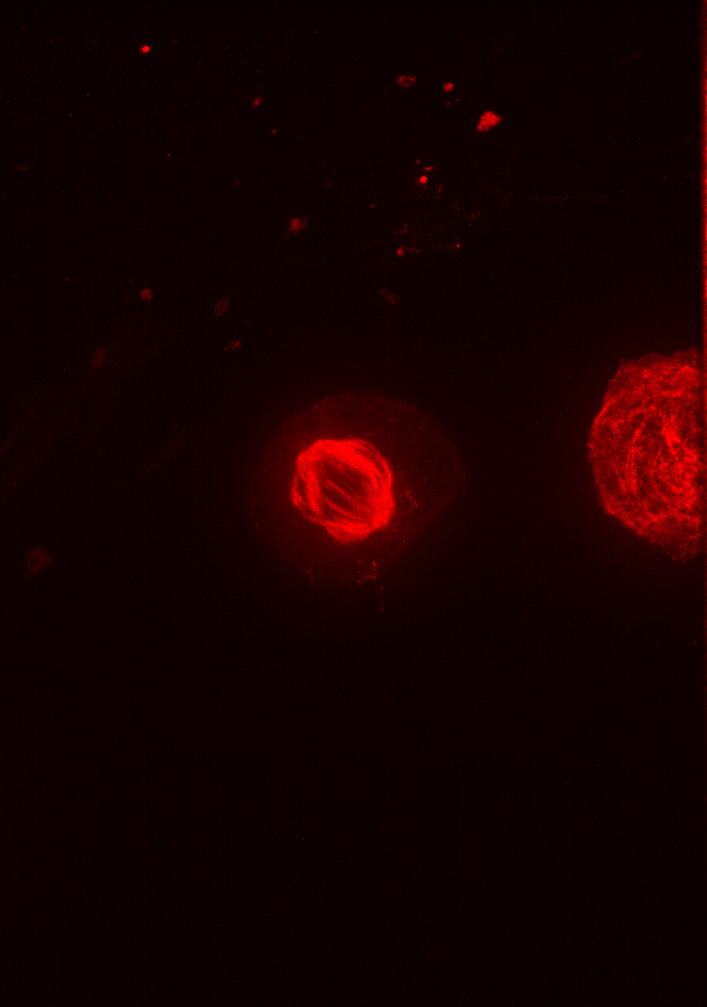

Supplement: Supplementary file 7 — Source data Fig. 5 [file 44318_2024_358_MOESM7_ESM.zip › Figure 5/F/C1-Metaphase-1.tif]

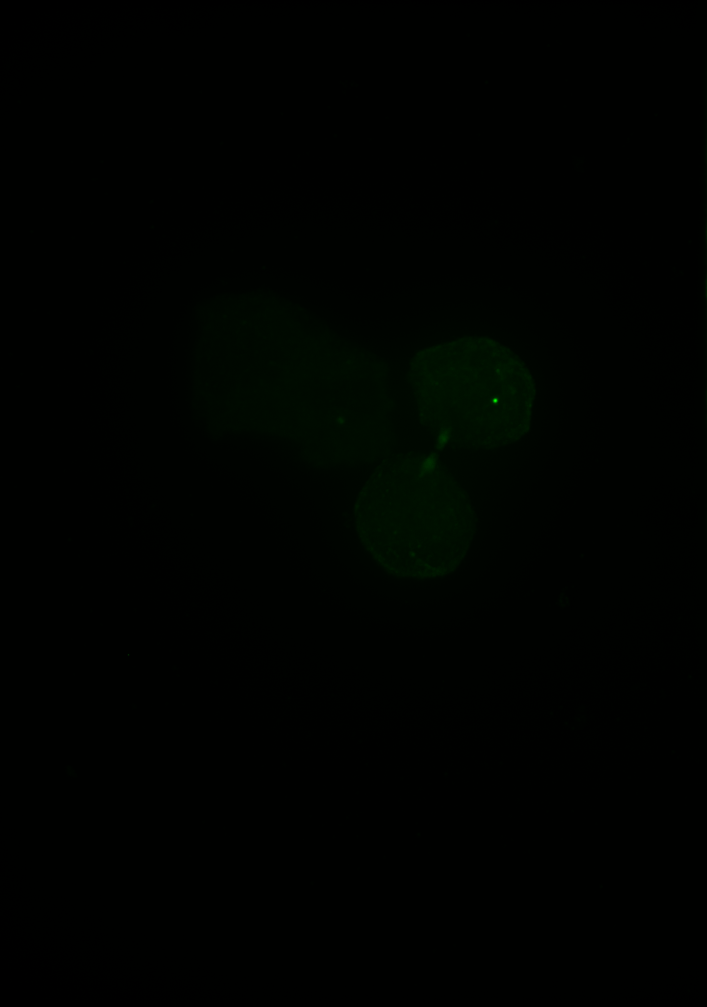

Supplement: Supplementary file 7 — Source data Fig. 5 [file 44318_2024_358_MOESM7_ESM.zip › Figure 5/F/C2-Anaphase-1.tif]

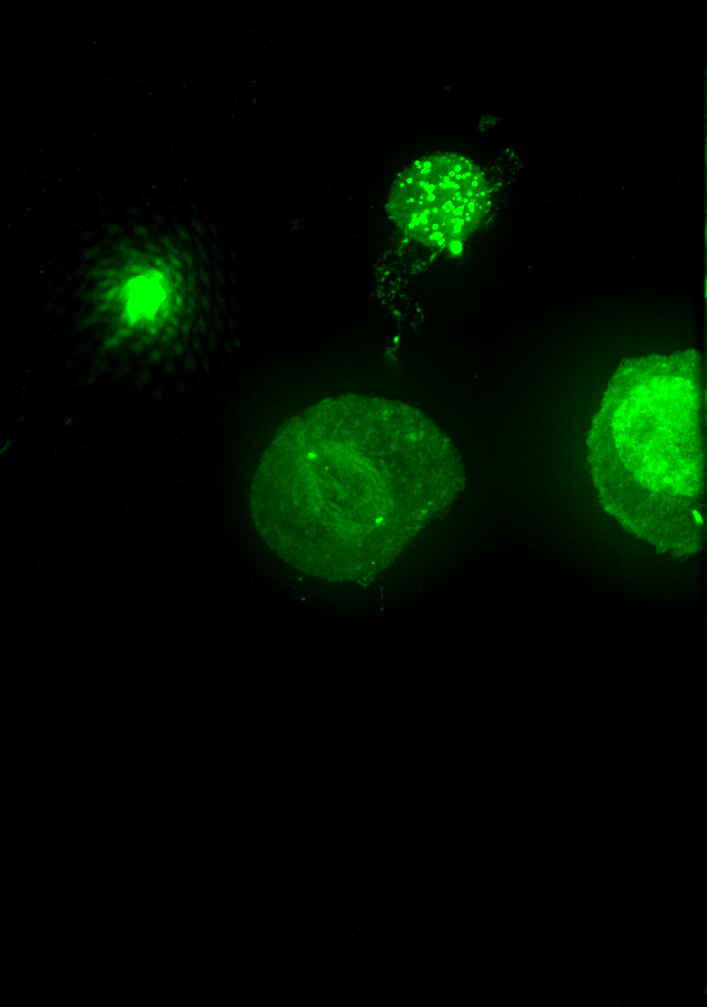

Supplement: Supplementary file 7 — Source data Fig. 5 [file 44318_2024_358_MOESM7_ESM.zip › Figure 5/F/C2-Metaphase-1.tif]

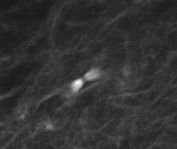

Supplement: Supplementary file 7 — Source data Fig. 5 [file 44318_2024_358_MOESM7_ESM.zip › Figure 5/G/C1-anaphaseSOP-1.tif]

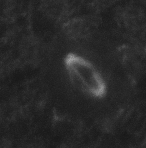

Supplement: Supplementary file 7 — Source data Fig. 5 [file 44318_2024_358_MOESM7_ESM.zip › Figure 5/G/C1-metaphaseSOP-1.tif]

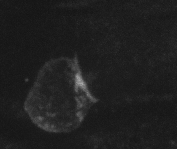

Supplement: Supplementary file 7 — Source data Fig. 5 [file 44318_2024_358_MOESM7_ESM.zip › Figure 5/G/C2-anaphaseSOP-1.tif]

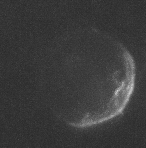

Supplement: Supplementary file 7 — Source data Fig. 5 [file 44318_2024_358_MOESM7_ESM.zip › Figure 5/G/C2-metaphaseSOP-1.tif]

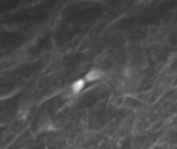

Supplement: Supplementary file 7 — Source data Fig. 5 [file 44318_2024_358_MOESM7_ESM.zip › Figure 5/G/C3-anaphaseSOP-1.tif]

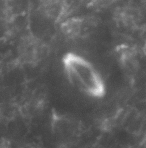

Supplement: Supplementary file 7 — Source data Fig. 5 [file 44318_2024_358_MOESM7_ESM.zip › Figure 5/G/C3-metaphaseSOP-1.tif]

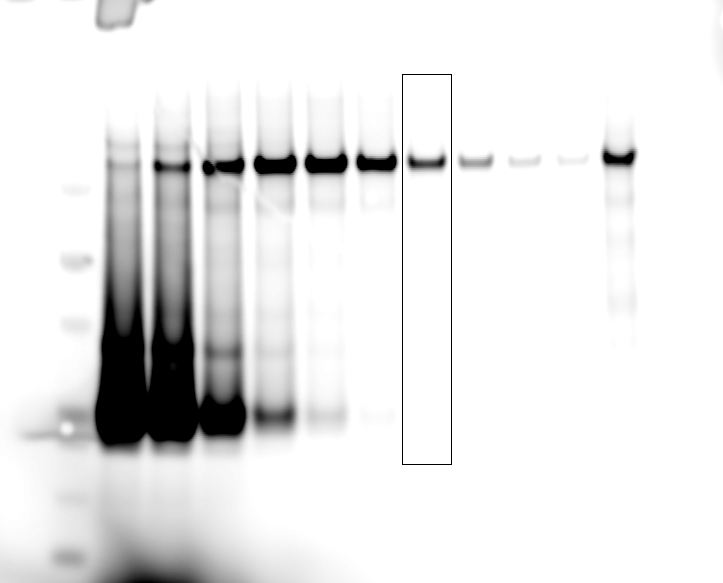

Supplement: Supplementary file 8 — Figure EV1 Source Data [file 44318_2024_358_MOESM8_ESM.zip › Figure EV1/A/488 fluorescence.tif]

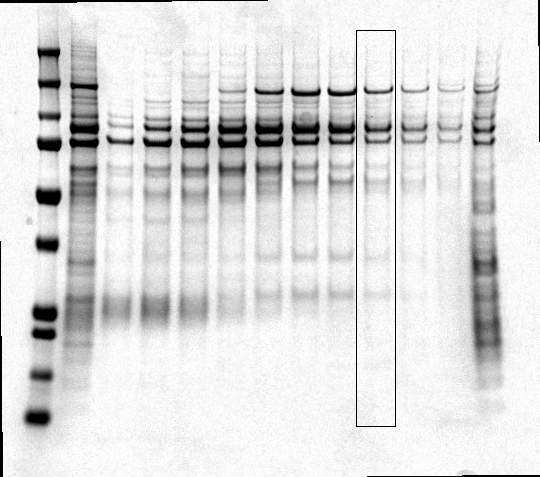

Supplement: Supplementary file 8 — Figure EV1 Source Data [file 44318_2024_358_MOESM8_ESM.zip › Figure EV1/A/coomassie.tif]

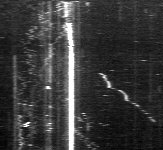

Supplement: Supplementary file 8 — Figure EV1 Source Data [file 44318_2024_358_MOESM8_ESM.zip › Figure EV1/B/C1-Kymograph-1.tif]

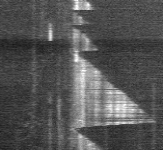

Supplement: Supplementary file 8 — Figure EV1 Source Data [file 44318_2024_358_MOESM8_ESM.zip › Figure EV1/B/C2-Kymograph-1.tif]

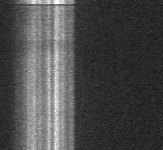

Supplement: Supplementary file 8 — Figure EV1 Source Data [file 44318_2024_358_MOESM8_ESM.zip › Figure EV1/B/C3-Kymograph-1.tif]

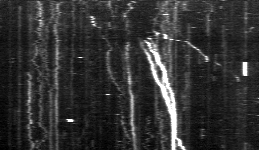

Supplement: Supplementary file 8 — Figure EV1 Source Data [file 44318_2024_358_MOESM8_ESM.zip › Figure EV1/C/C1-Kymograph-1.tif]

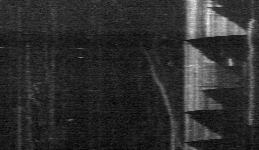

Supplement: Supplementary file 8 — Figure EV1 Source Data [file 44318_2024_358_MOESM8_ESM.zip › Figure EV1/C/C2-Kymograph-1.tif]

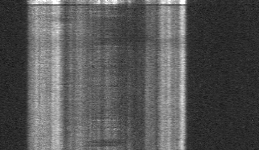

Supplement: Supplementary file 8 — Figure EV1 Source Data [file 44318_2024_358_MOESM8_ESM.zip › Figure EV1/C/C3-Kymograph-1.tif]

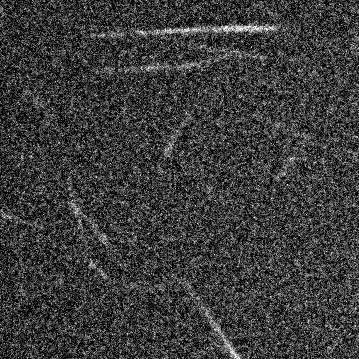

Supplement: Supplementary file 9 — Figure EV2 Source Data [file 44318_2024_358_MOESM9_ESM.zip › Figure EV2/A/C1-Fourth row-1.tif]

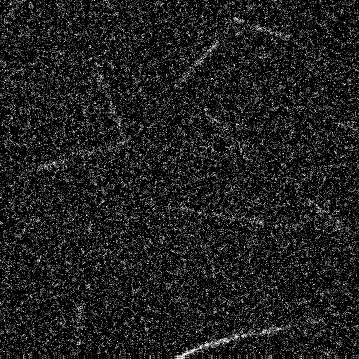

Supplement: Supplementary file 9 — Figure EV2 Source Data [file 44318_2024_358_MOESM9_ESM.zip › Figure EV2/A/C1-Second row-1.tif]

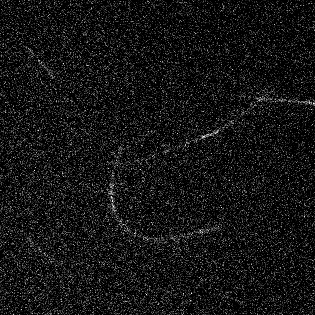

Supplement: Supplementary file 9 — Figure EV2 Source Data [file 44318_2024_358_MOESM9_ESM.zip › Figure EV2/A/C1-Third row-1.tif]

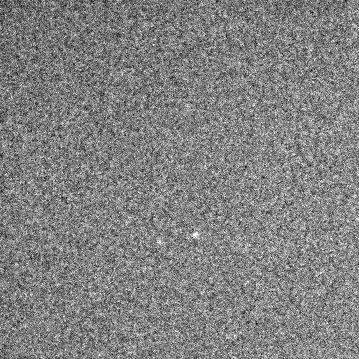

Supplement: Supplementary file 9 — Figure EV2 Source Data [file 44318_2024_358_MOESM9_ESM.zip › Figure EV2/A/C2-Fourth row-1.tif]

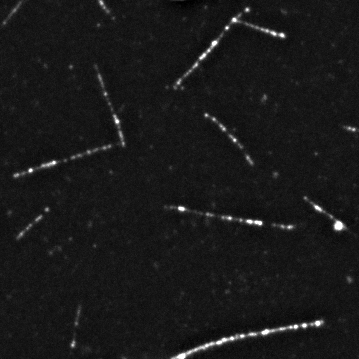

Supplement: Supplementary file 9 — Figure EV2 Source Data [file 44318_2024_358_MOESM9_ESM.zip › Figure EV2/A/C2-Second row-1.tif]

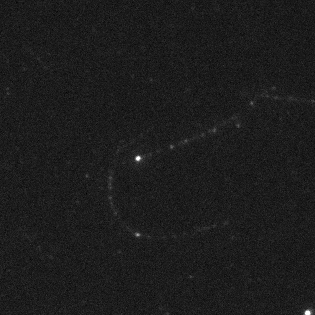

Supplement: Supplementary file 9 — Figure EV2 Source Data [file 44318_2024_358_MOESM9_ESM.zip › Figure EV2/A/C2-Third row-1.tif]

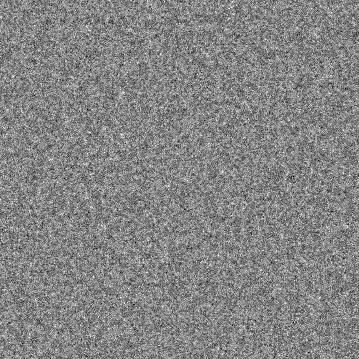

Supplement: Supplementary file 9 — Figure EV2 Source Data [file 44318_2024_358_MOESM9_ESM.zip › Figure EV2/A/C3-Second row-1.tif]

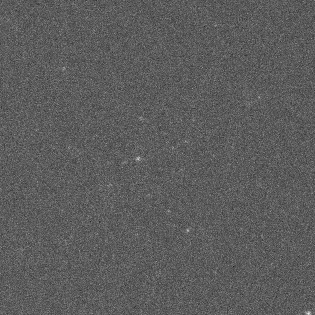

Supplement: Supplementary file 9 — Figure EV2 Source Data [file 44318_2024_358_MOESM9_ESM.zip › Figure EV2/A/C3-Third row-1.tif]

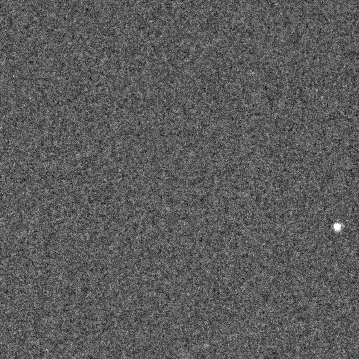

Supplement: Supplementary file 9 — Figure EV2 Source Data [file 44318_2024_358_MOESM9_ESM.zip › Figure EV2/A/C4-Second row-1.tif]

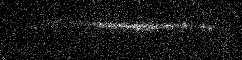

Supplement: Supplementary file 9 — Figure EV2 Source Data [file 44318_2024_358_MOESM9_ESM.zip › Figure EV2/C/C1-C-1.tif]

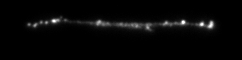

Supplement: Supplementary file 9 — Figure EV2 Source Data [file 44318_2024_358_MOESM9_ESM.zip › Figure EV2/C/C2-C-1.tif]

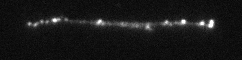

Supplement: Supplementary file 9 — Figure EV2 Source Data [file 44318_2024_358_MOESM9_ESM.zip › Figure EV2/C/C3-C-1.tif]

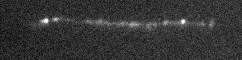

Supplement: Supplementary file 9 — Figure EV2 Source Data [file 44318_2024_358_MOESM9_ESM.zip › Figure EV2/C/C4-C-1.tif]

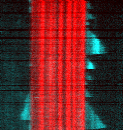

Supplement: Supplementary file 10 — Figure EV3 Source Data [file 44318_2024_358_MOESM10_ESM.zip › Figure EV3/A/Elongator-Kymo.tif]

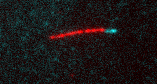

Supplement: Supplementary file 10 — Figure EV3 Source Data [file 44318_2024_358_MOESM10_ESM.zip › Figure EV3/A/Elongator-Still.tif]

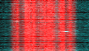

Supplement: Supplementary file 10 — Figure EV3 Source Data [file 44318_2024_358_MOESM10_ESM.zip › Figure EV3/A/Tubulin-control-Kymo.tif]

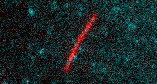

Supplement: Supplementary file 10 — Figure EV3 Source Data [file 44318_2024_358_MOESM10_ESM.zip › Figure EV3/A/Tubulin-control-Still.tif]

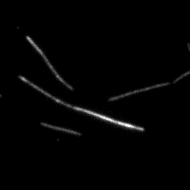

Supplement: Supplementary file 10 — Figure EV3 Source Data [file 44318_2024_358_MOESM10_ESM.zip › Figure EV3/D/D.tif]

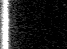

Supplement: Supplementary file 10 — Figure EV3 Source Data [file 44318_2024_358_MOESM10_ESM.zip › Figure EV3/E/E.tif]

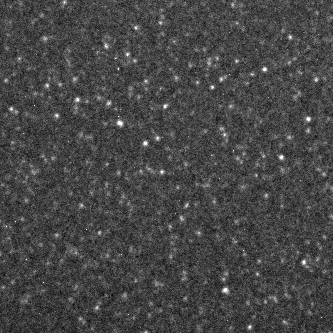

Supplement: Supplementary file 10 — Figure EV3 Source Data [file 44318_2024_358_MOESM10_ESM.zip › Figure EV3/F/S2-Elongator-Ctrl.tif]

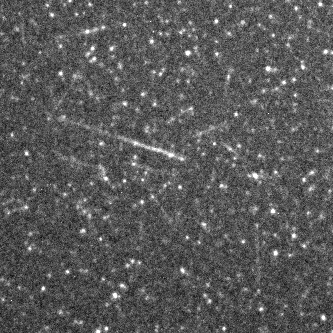

Supplement: Supplementary file 10 — Figure EV3 Source Data [file 44318_2024_358_MOESM10_ESM.zip › Figure EV3/F/S2-Elongator-SNAP-Elp3.tif]

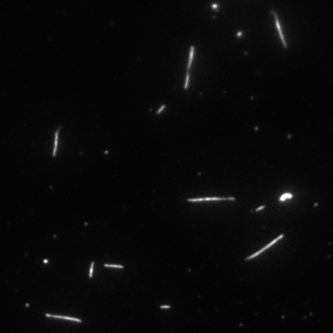

Supplement: Supplementary file 10 — Figure EV3 Source Data [file 44318_2024_358_MOESM10_ESM.zip › Figure EV3/F/S2-Microtubule-Ctrl.tif]

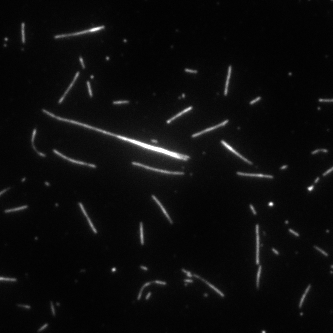

Supplement: Supplementary file 10 — Figure EV3 Source Data [file 44318_2024_358_MOESM10_ESM.zip › Figure EV3/F/S2-Microtubule-SNAP-Elp3.tif]

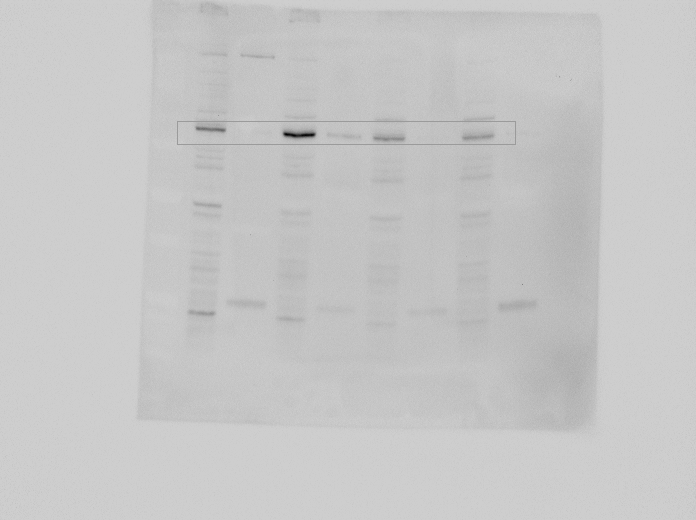

Supplement: Supplementary file 11 — Figure EV4 Source Data [file 44318_2024_358_MOESM11_ESM.zip › Figure EV4/A/Elp2 blot.tif]

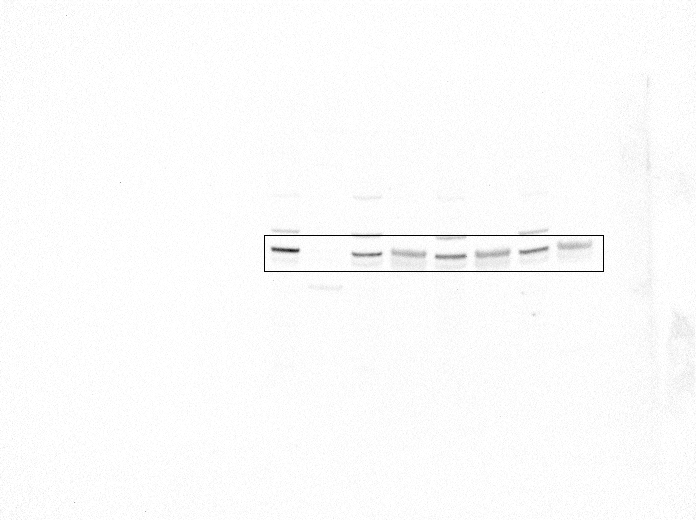

Supplement: Supplementary file 11 — Figure EV4 Source Data [file 44318_2024_358_MOESM11_ESM.zip › Figure EV4/A/Elp4 blot.tif]

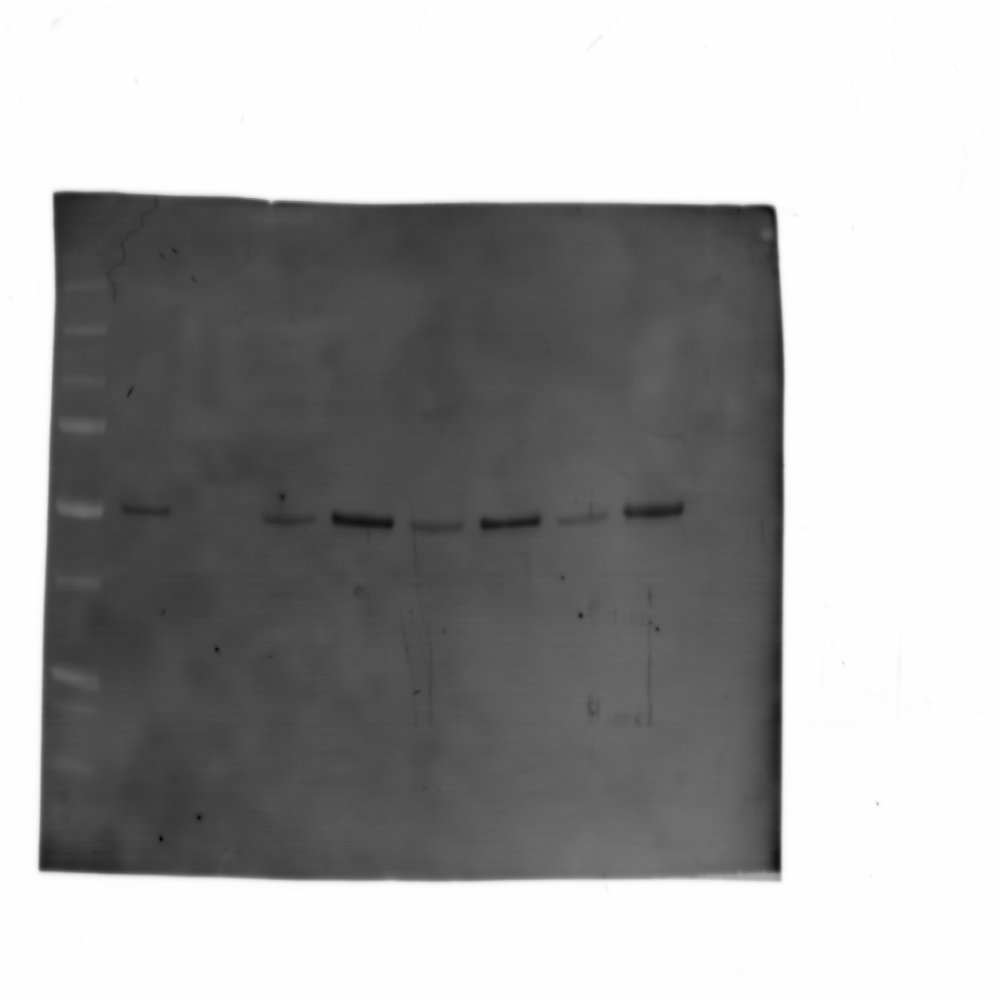

Supplement: Supplementary file 11 — Figure EV4 Source Data [file 44318_2024_358_MOESM11_ESM.zip › Figure EV4/A/tubulin blot.tif]

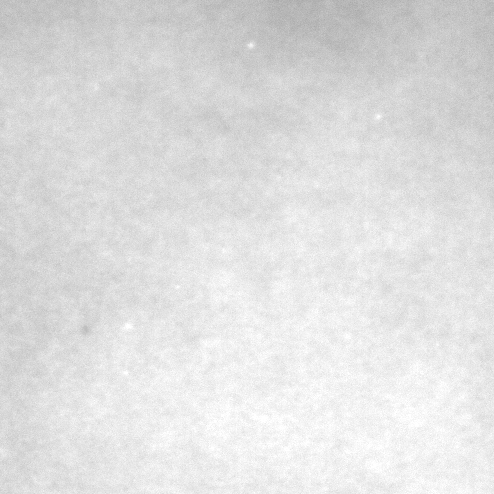

Supplement: Supplementary file 11 — Figure EV4 Source Data [file 44318_2024_358_MOESM11_ESM.zip › Figure EV4/C/First column.tif]

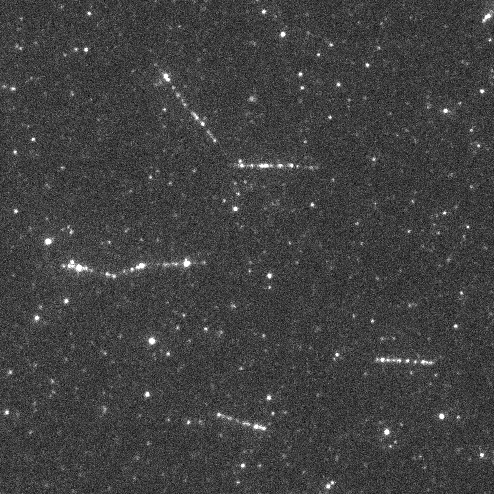

Supplement: Supplementary file 11 — Figure EV4 Source Data [file 44318_2024_358_MOESM11_ESM.zip › Figure EV4/C/Fourth column.tif]

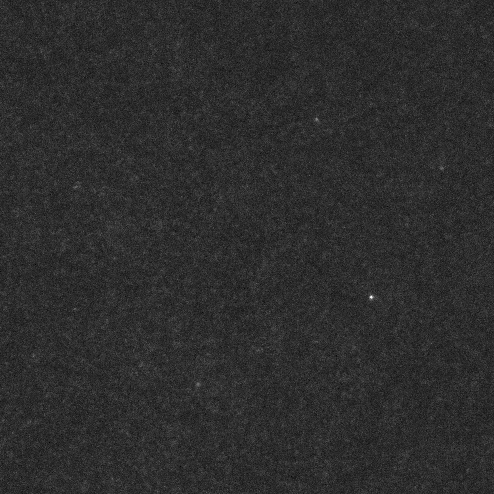

Supplement: Supplementary file 11 — Figure EV4 Source Data [file 44318_2024_358_MOESM11_ESM.zip › Figure EV4/C/Second column.tif]

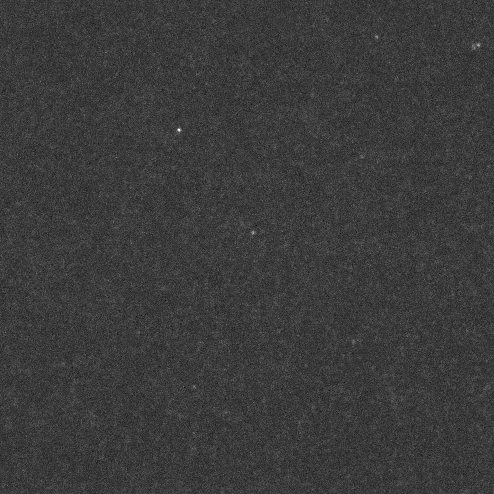

Supplement: Supplementary file 11 — Figure EV4 Source Data [file 44318_2024_358_MOESM11_ESM.zip › Figure EV4/C/Third column.tif]

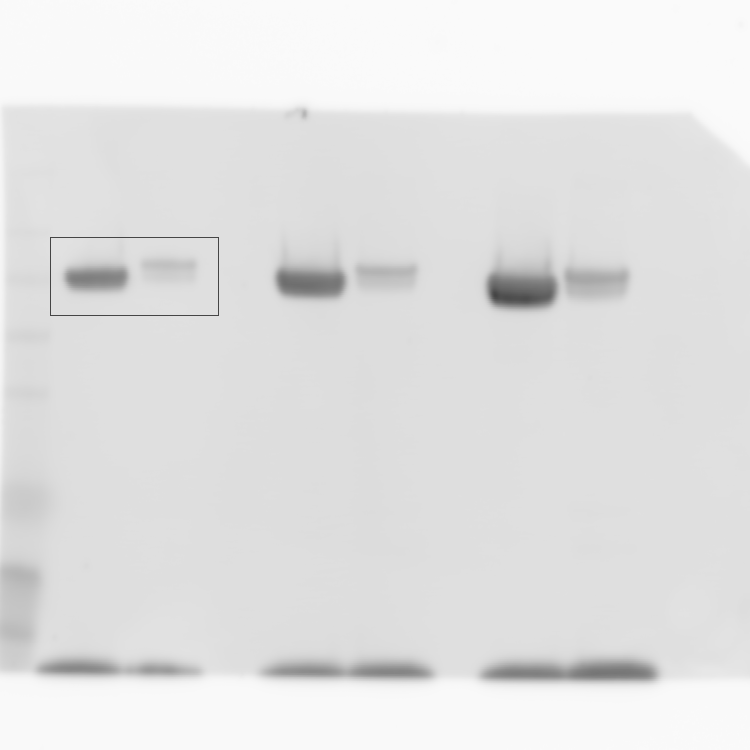

Supplement: Supplementary file 12 — Figure EV5 Source Data [file 44318_2024_358_MOESM12_ESM.zip › Figure EV5/B/K40Ac blot.tif]

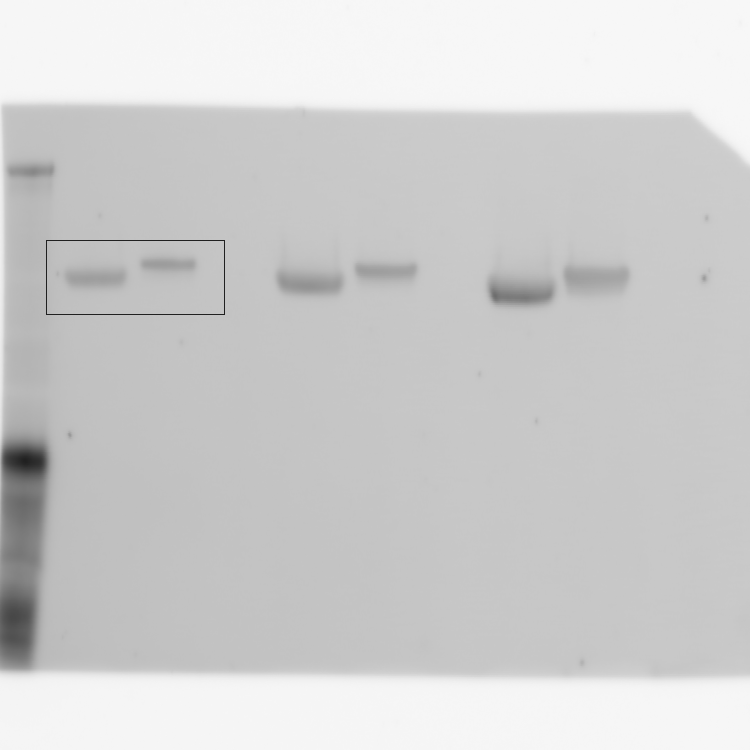

Supplement: Supplementary file 12 — Figure EV5 Source Data [file 44318_2024_358_MOESM12_ESM.zip › Figure EV5/B/polyGlu blot.tif]

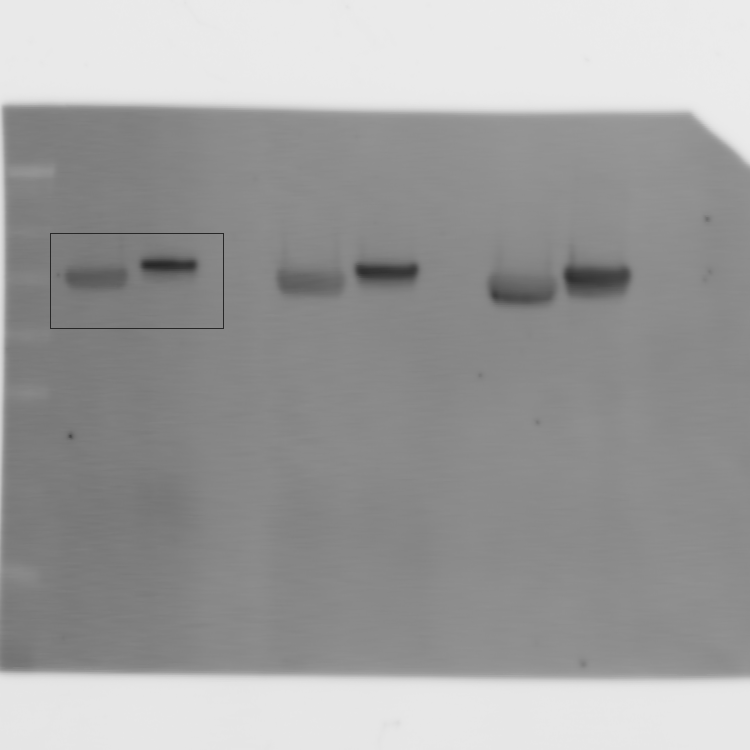

Supplement: Supplementary file 12 — Figure EV5 Source Data [file 44318_2024_358_MOESM12_ESM.zip › Figure EV5/B/Tubulin blot.tif]

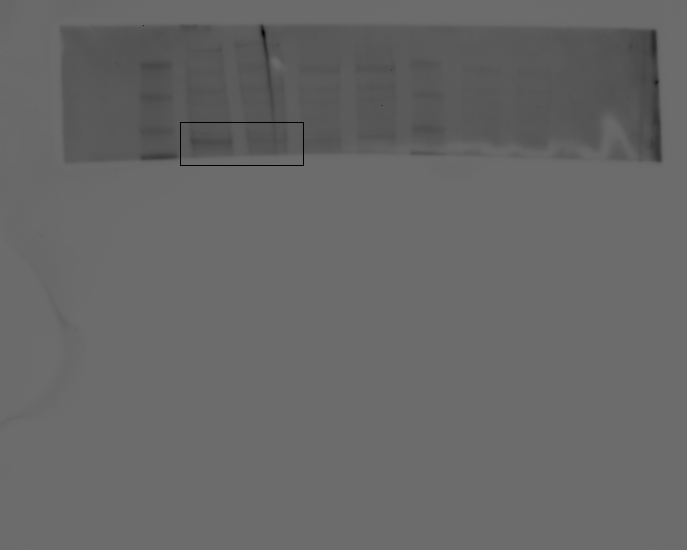

Supplement: Supplementary file 12 — Figure EV5 Source Data [file 44318_2024_358_MOESM12_ESM.zip › Figure EV5/C/Elp2 blot.tif]

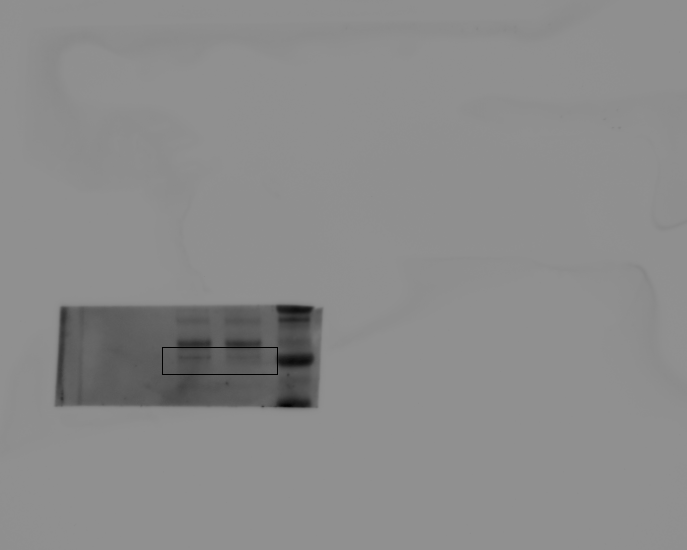

Supplement: Supplementary file 12 — Figure EV5 Source Data [file 44318_2024_358_MOESM12_ESM.zip › Figure EV5/C/Elp4 blot.tif]

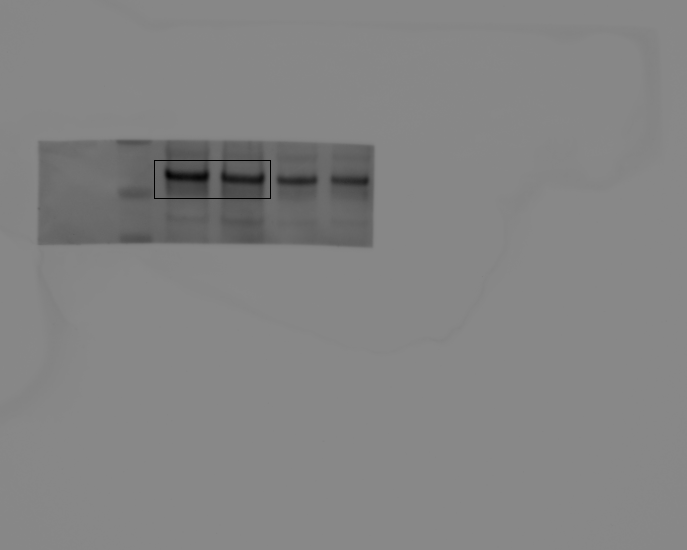

Supplement: Supplementary file 12 — Figure EV5 Source Data [file 44318_2024_358_MOESM12_ESM.zip › Figure EV5/C/Tubulin blot.tif]

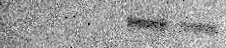

Supplement: Supplementary file 12 — Figure EV5 Source Data [file 44318_2024_358_MOESM12_ESM.zip › Figure EV5/D/Elp3-3T3.tif]

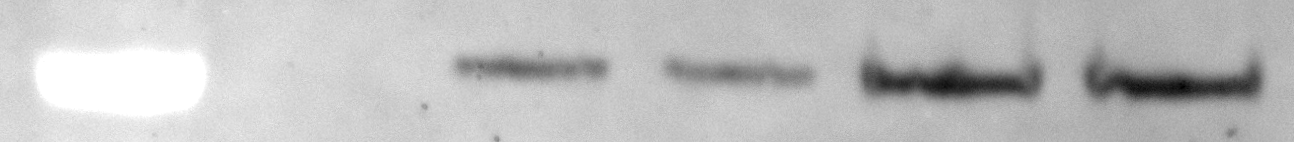

Supplement: Supplementary file 12 — Figure EV5 Source Data [file 44318_2024_358_MOESM12_ESM.zip › Figure EV5/D/TotTub-3T3.tif]
