# Supplementary figures and images for: Elongator is a microtubule polymerase selective for polyglutamylated tubulin (part 2 of 2)
Source: EMBO J. 2025 Jan 15;44(5):1322–53. doi: 10.1038/s44318-024-00358-0 (PMC11876699; doi:10.1038/s44318-024-00358-0)

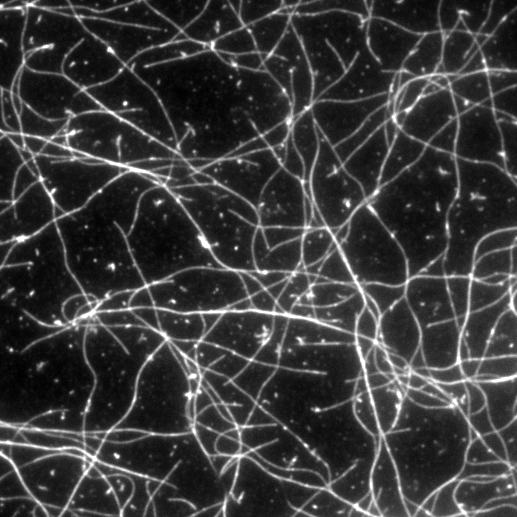

Supplement: Supplementary file 12 — Figure EV5 Source Data [file 44318_2024_358_MOESM12_ESM.zip › Figure EV5/E/C2-Pig tubulin control.tif]

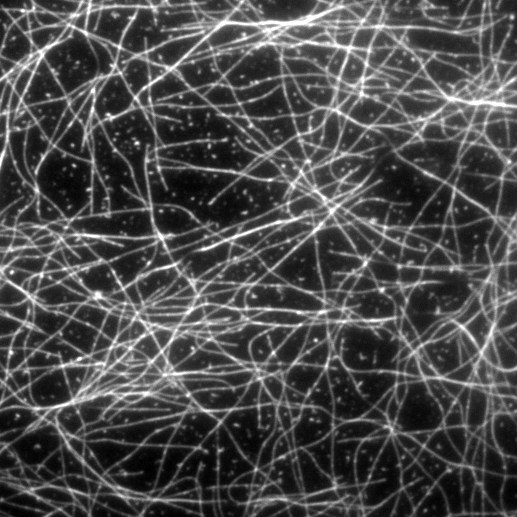

Supplement: Supplementary file 12 — Figure EV5 Source Data [file 44318_2024_358_MOESM12_ESM.zip › Figure EV5/E/C2-Pig tubulin Elongator.tif]

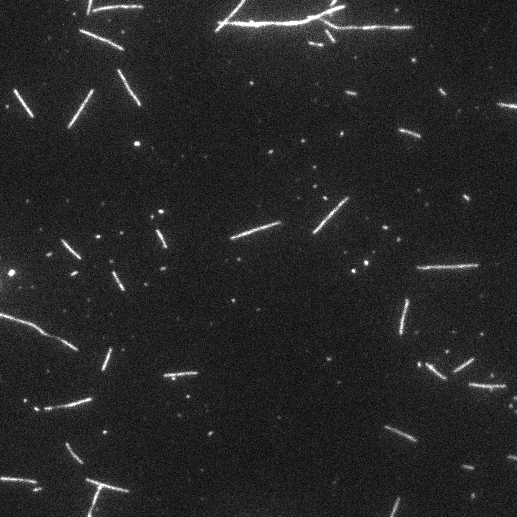

Supplement: Supplementary file 12 — Figure EV5 Source Data [file 44318_2024_358_MOESM12_ESM.zip › Figure EV5/E/Pig tubulin Elongator.tif]

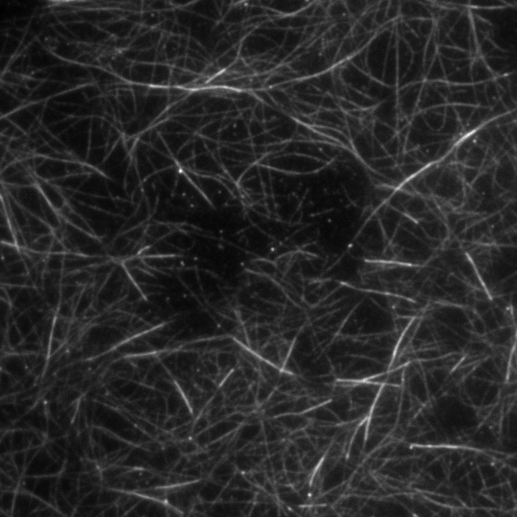

Supplement: Supplementary file 12 — Figure EV5 Source Data [file 44318_2024_358_MOESM12_ESM.zip › Figure EV5/G/C2-Hela-Control.tif]

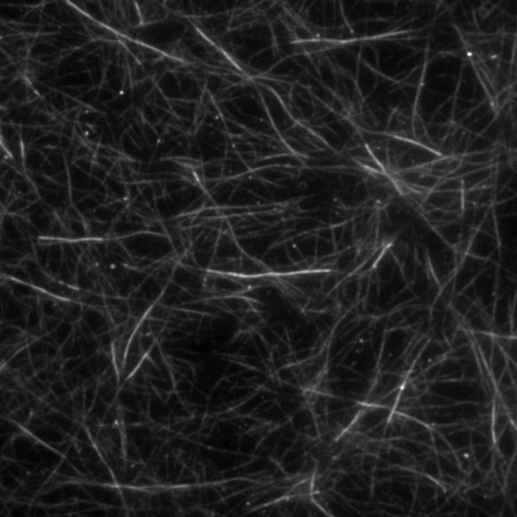

Supplement: Supplementary file 12 — Figure EV5 Source Data [file 44318_2024_358_MOESM12_ESM.zip › Figure EV5/G/C2-HeLa-Elongator.tif]

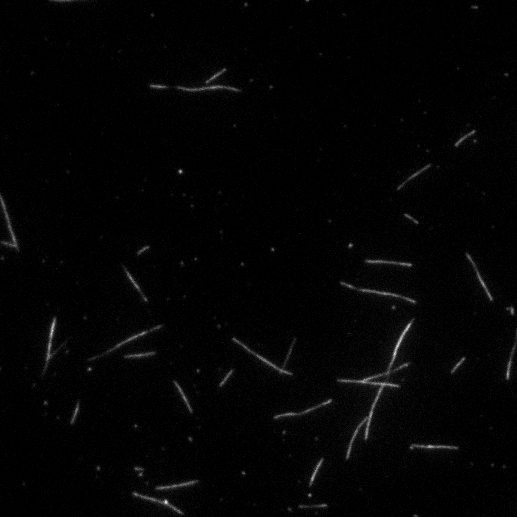

Supplement: Supplementary file 12 — Figure EV5 Source Data [file 44318_2024_358_MOESM12_ESM.zip › Figure EV5/G/Hela-Control.tif]

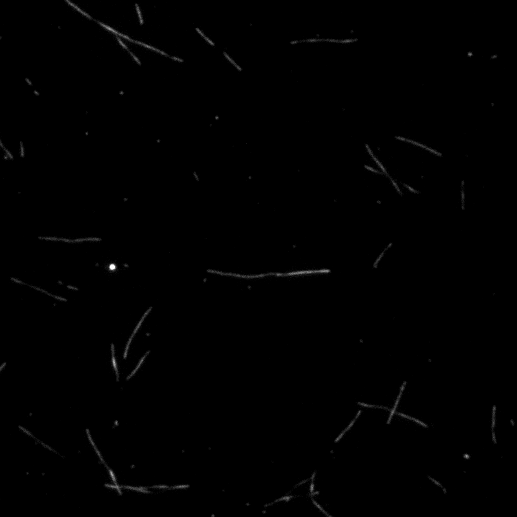

Supplement: Supplementary file 12 — Figure EV5 Source Data [file 44318_2024_358_MOESM12_ESM.zip › Figure EV5/G/HeLa-Elongator.tif]
