# Supplementary material for: Elongator is a microtubule polymerase selective for polyglutamylated tubulin
Source: EMBO J. 2025 Jan 15;44(5):1322–53. doi: 10.1038/s44318-024-00358-0 (PMC11876699; doi:10.1038/s44318-024-00358-0)
Supplement: Supplementary file 13 — Expanded View Figures [file 44318_2024_358_MOESM13_ESM.pdf]

Expanded View Figures

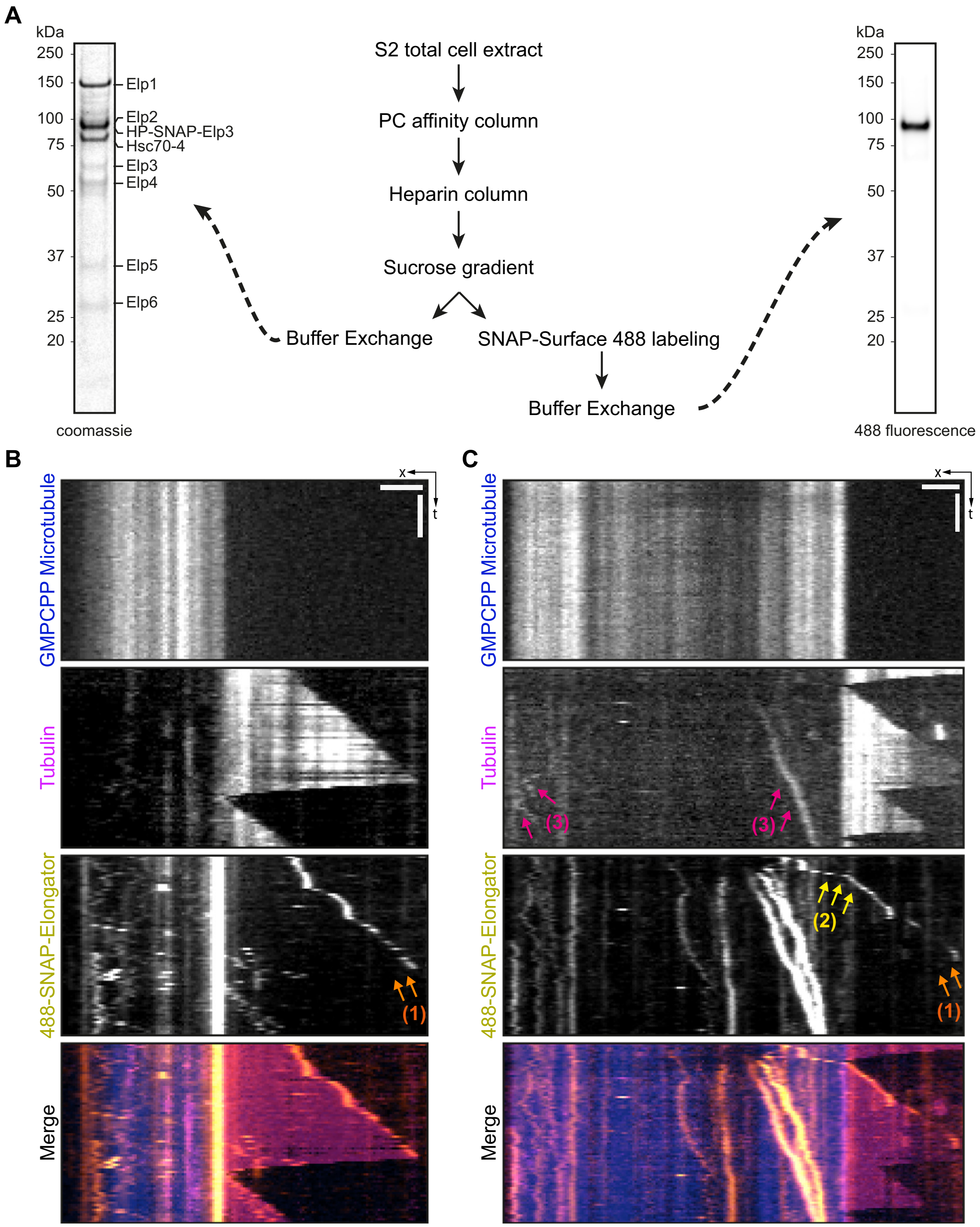

Figure EV1. Elongator purification and characterization.

(**A**) Purification and fluorescent labelling of Elongator complex from *Drosophila* S2 cells (see methods). (**B, C**) Biotinylated, rhodamine-labelled GMPCPP-stabilized seeds (red) are anchored via NeutrAvidin to PLL-PEG-Silane-Biotin. Free tubulin (10% HiLyte 647-labelled, cyan) and Alexa488-SNAP-Elongator (yellow) is added and imaged by TIRFM. Several behaviors of the Elongator complex can be observed: (1) Elongator detaches from the microtubule ends when microtubules undergo catastrophe (orange arrows); (2) Elongator can "jump" from the GMPCPP seed to the dynamic microtubule, tracking the growing end (yellow arrows); (3) Tubulin signal can be observed diffusing together with Elongator (magenta arrows). Note that 488-Elongator signal can also be observed diffusing on the microtubules. Scale bars = 2 min/2 µm. Source data are available online for this figure.

© The Author(s)

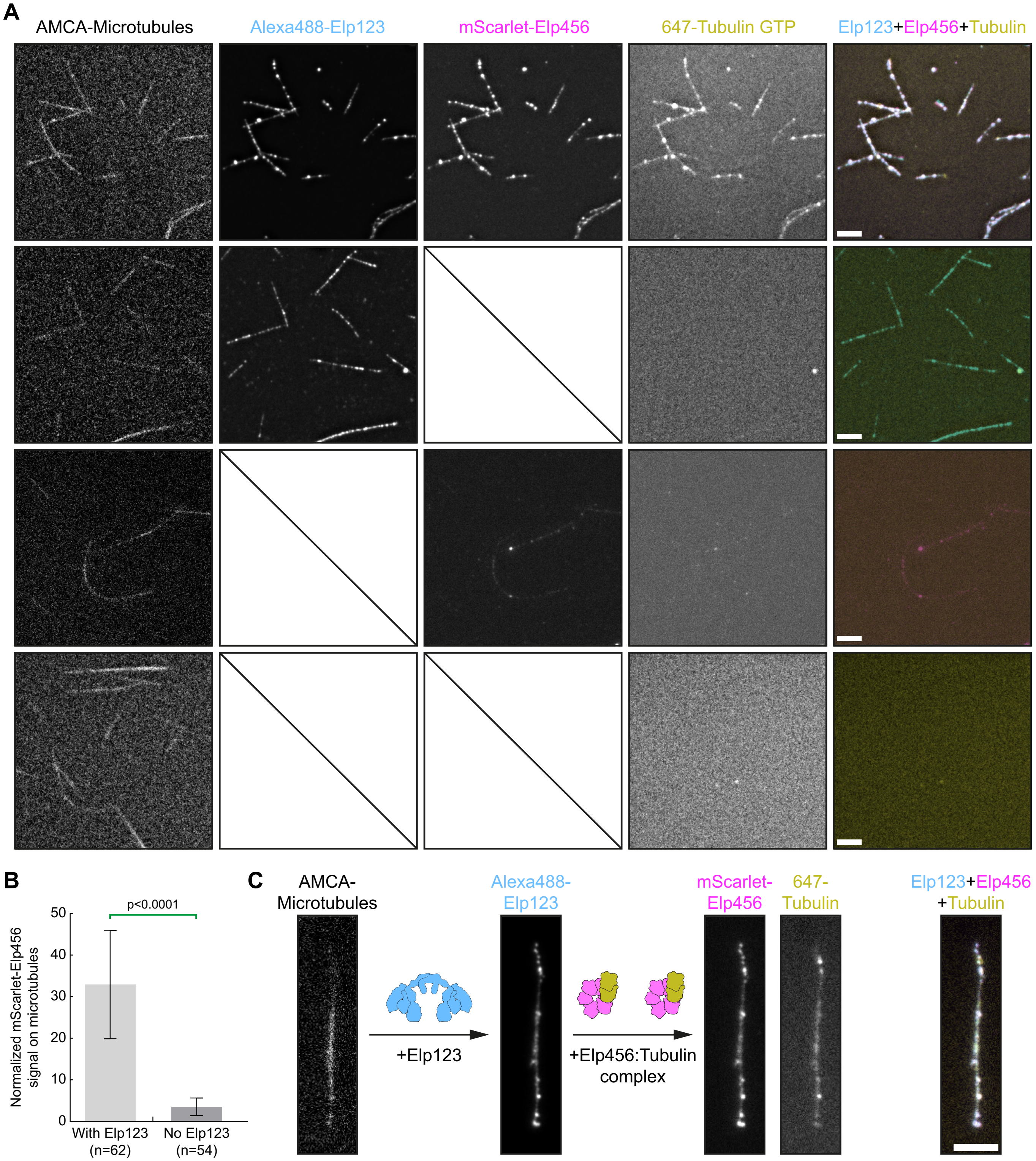

Figure EV2. Elongator-tubulin complex reconstitution on microtubules.

(**A**) Controls for Fig. 4A. Note that the first row is duplicated from Fig. 4A shown here for convenience. In the absence of Elp456, no tubulin signal can be detected on microtubules (second row). Similarly, in the absence of both Elp123 and Elp456, no tubulin signal is detected on microtubules (bottom row). Note that in the absence of Elp123, a weak mScarlet-Elp456 signal can be observed on microtubules (third row). (**B**) This signal is however significantly weaker than when Elp123 is also present ($P < 0.0001$ two-tailed, unpaired t-test). Error bars are mean ± standard deviation for quantified intensities. *n*, quantified microtubules. (**C**) A pre-formed mScarlet-Elp456:647-Tubulin complex can be recruited to Elp123-decorated microtubules. Scale bars = 5 µm. Source data are available online for this figure.

 © The Author(s)

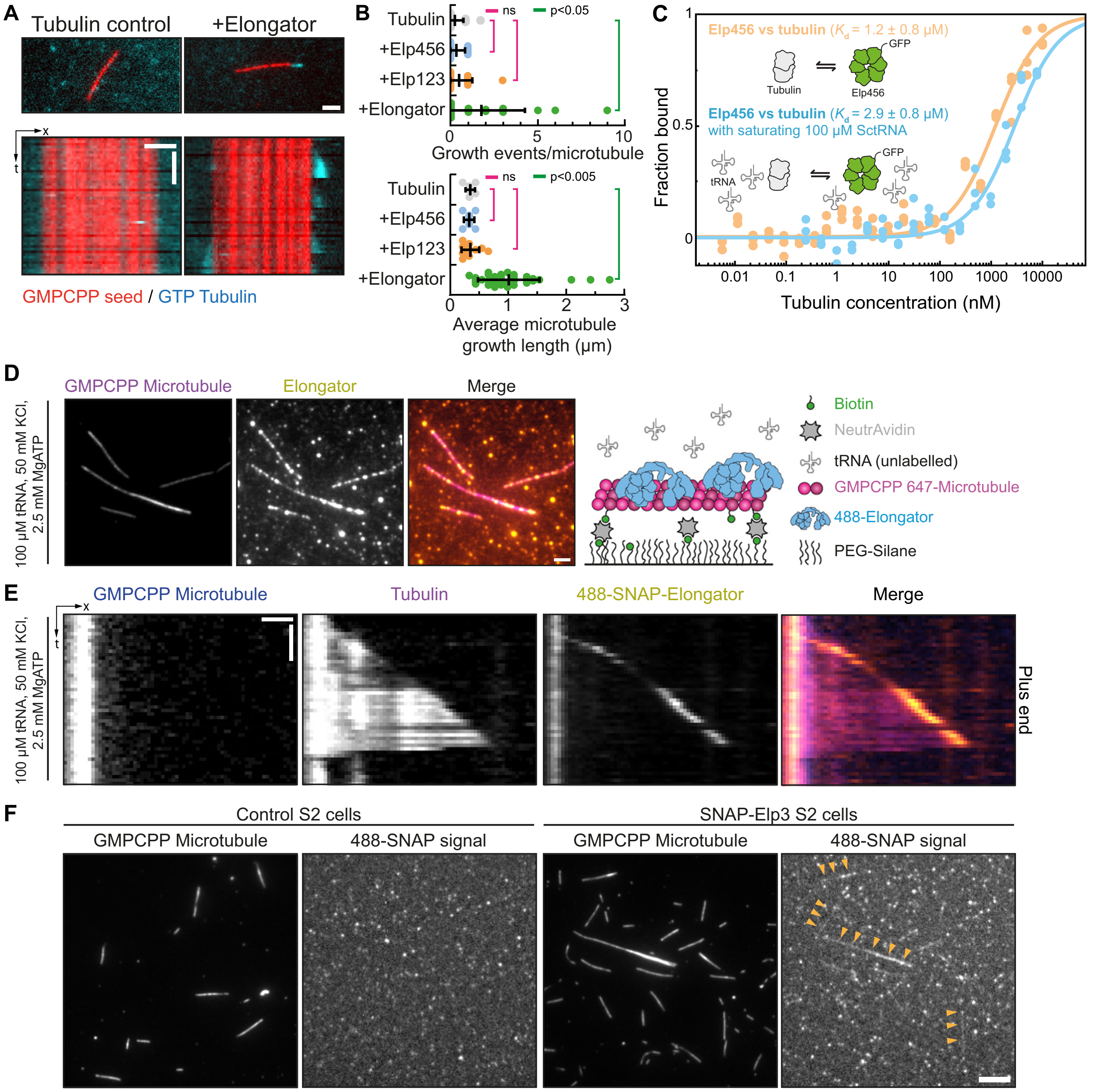
A
Tubulin control
+Elongator
x
t
GMPCPP seed / GTP Tubulin
B
Tubulin
+Elp456
+Elp123
+Elongator
ns
p<0.05
0 5 10
Growth events/microtubule
p<0.005
0 1 2 3
Average microtubule growth length (µm)
C
Elp456 vs tubulin (Kd = 1.2 ± 0.8 µM)
Elp456 vs tubulin (Kd = 2.9 ± 0.8 µM) with saturating 100 µM SctRNA
Tubulin
Elp456
GFP
tRNA
Fraction bound
0 0.5 1
0.01 0.1 1 10 100 1000 10000
Tubulin concentration (nM)
D
GMPCPP Microtubule
Elongator
Merge
100 µM tRNA, 50 mM KCl, 2.5 mM MgATP
Biotin
NeutrAvidin
tRNA (unlabelled)
GMPCPP 647-Microtubule
488-Elongator
PEG-Silane
E
GMPCPP Microtubule
Tubulin
488-SNAP-Elongator
Merge
Plus end
F
Control S2 cells
SNAP-Elp3 S2 cells
GMPCPP Microtubule
488-SNAP signal

© The Author(s)

Figure EV3. Elongator can discriminate between microtubules and tRNA.

(**A, B**) Elongator decreases the effective critical concentration of tubulin for microtubule elongation. (**A**) Rhodamine-labelled GMPCPP stabilized microtubules incubated with GTP-tubulin near the critical concentration (6 µM) and imaged by TIRFM in the presence or absence of Elongator. (**B**) Quantification of the number of microtubule growth events observed, and their length, in (**A**). (Upper panel) n = quantified microtubules = 20. P values were calculated for a two-tailed Kruskal–Wallis test followed by Dunn's multiple comparison test. Exact P values: Tubulin vs Elp456 > 0.9999, Tubulin vs Elp123 > 0.9999, Tubulin vs Elongator 0.0320. (Lower panel) n = observed growth length in the growth events quantified for the upper panel = 6 (Tubulin), 7 (+Elp456), 10 (+Elp123) and 36 (+ Elongator). P values were calculated for an Ordinary one-way ANOVA test followed by two-tailed Turkey multiple comparison. Exact P values: Tubulin vs Elp456 > 0.9999, Tubulin vs Elp123 0.9995, Tubulin vs Elongator 0.0024. Elongator concentrations used are as in Fig. 4. (**C**) Measurement of the affinity between tubulin and eGFP-Elp456 in high tRNA buffer (100 µM tRNA, 2.5 mM MgATP) using microscale thermophoresis (see "Methods"). Estimated dissociation constant (K_d) values are indicated (mean ± s.d.; n = 3). Note that the "Elp456 vs tubulin" dataset is the same as the one presented in Fig. 5A, shown here for convenience. (**D**) Rhodamine-labelled GMPCPP stabilized microtubules incubated with 25 nM SNAP-Elongator labelled with AlexaFluor488 dye in a buffer containing 100 µM tRNA, 50 mM KCl and 2.5 mM MgADP and observed by TIRFM. Salt and high tRNA concentrations do not prevent microtubule binding by Elongator. (**E**) Representative kymograph showing Alexa488 SNAP-Elongator complex tracking the plus end of a microtubule in the same conditions as in (**D**). (**F**) Total cell extracts of *Drosophila* S2 cells incubated with rhodamine-labelled GMPCPP stabilized microtubules and Alexa-488 SNAP ligand . In non-transfected cells extracts (left) no signal can be detected on microtubules. In extracts of cells expressing SNAP-Elp3 (see methods), clear signal can be observed on microtubules (orange arrows). Scale bars = 2 µm (**A**, **D**), 2 min/2 µm (**A**, **E**), 5 µm (**F**). Source data are available online for this figure.

 © The Author(s)

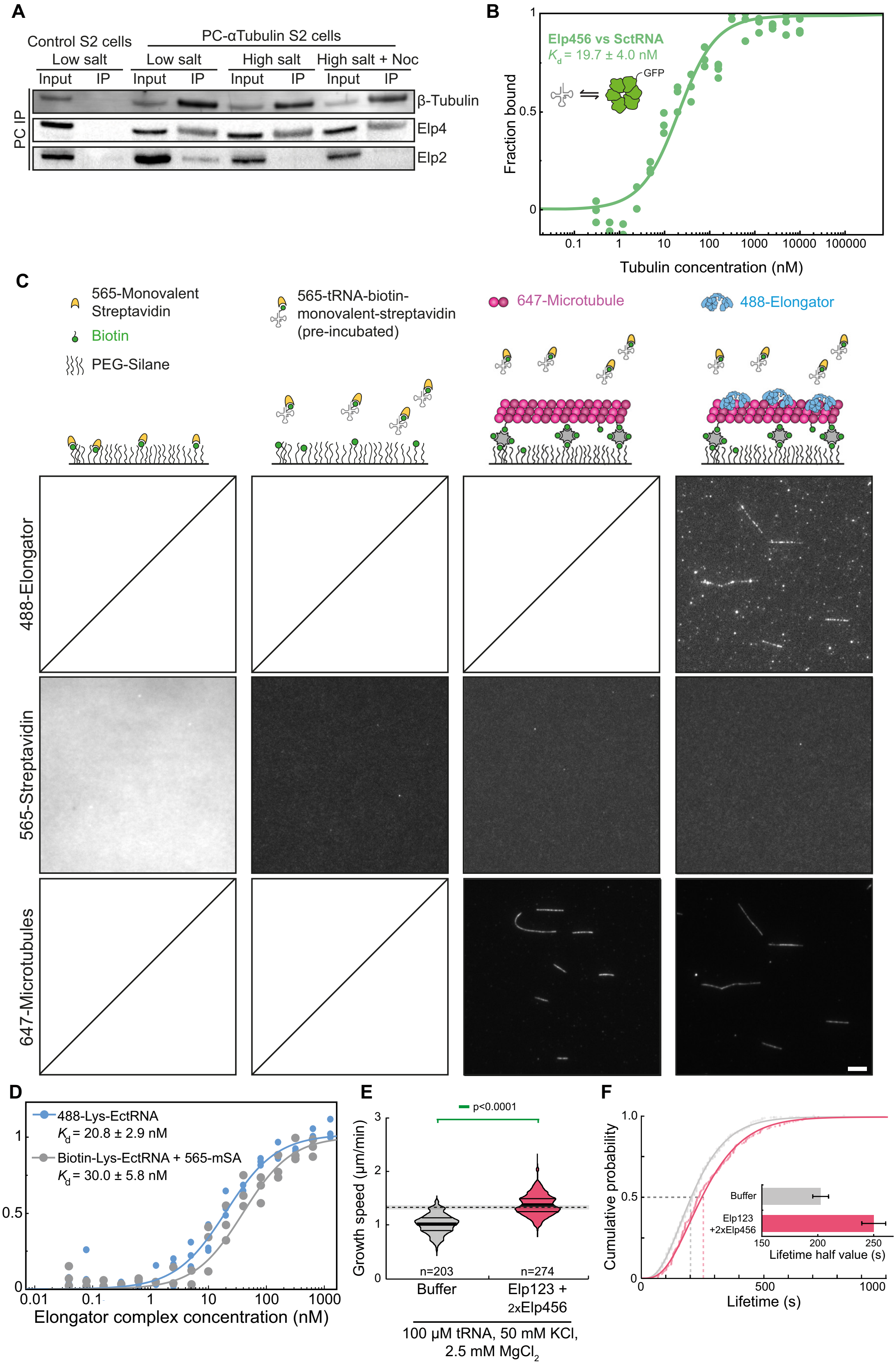
A
Control S2 cells
PC-αTubulin S2 cells
Low salt
Low salt
High salt
High salt + Noc
Input
IP
PC IP
β-Tubulin
Elp4
Elp2
B
Elp456 vs SctRNA
K_d = 19.7 ± 4.0 nM
GFP
Fraction bound
Tubulin concentration (nM)
C
565-Monovalent Streptavidin
Biotin
PEG-Silane
565-tRNA-biotin-monovalent-streptavidin (pre-incubated)
647-Microtubule
488-Elongator
488-Elongator
565-Streptavidin
647-Microtubules
D
488-Lys-EctRNA
K_d = 20.8 ± 2.9 nM
Biotin-Lys-EctRNA + 565-mSA
K_d = 30.0 ± 5.8 nM
Elongator complex concentration (nM)
E
p<0.0001
Growth speed (µm/min)
n=203
n=274
Buffer
Elp123 + 2xElp456
100 µM tRNA, 50 mM KCl, 2.5 mM $MgCl_2$
F
Cumulative probability
Buffer
Elp123 +2xElp456
Lifetime half value (s)
Lifetime (s)

Figure EV4. Elongator does not bind to tRNA while bound to microtubules.

(**A**) PC-tag immunoprecipitation of PC-α-Tubulin from S2 cells expressing PC-α-Tubulin. Low salt: 50 mM KCl. High salt: 300 mM KCl. Note that his PC-tag immunoprecipitation is performed at 4 °C and in the presence of 1 mM $CaCl_2$, conditions which are well established to depolymerize microtubules and therefore lead to tubulin dimers rather than microtubules. (**B**) Measurement of the binding affinity between eGFP-Elp456 and tRNA (total tRNA from *S. cerevisiae*). Calculated dissociation constant (K_d) values are indicated (mean ± s.d.; $n = 3$). (**C**) (First column) ATTO565 labelled monovalent streptavidin (mSA2-565) is functional and binds to the PEG-Silane-Biotin. (Second column) mSA2-565 saturated with tRNA-biotin (Promega) using a 10:1 ratio (tRNA:mSA2-565) does not bind to the surface. (Third column) tRNA-biotin-mSA2-565 (10:1 ratio as in the second column) does not bind rhodamine-labelled, biotinylated GMPCPP-microtubules attached to the surface via neutravidin. Note that after the addition of microtubules, a solution of 500 μg/ml biotin was added in the chamber to quench the neutravidin. Then, the tRNA-biotin-mSA2-565 mixture was added. (Fourth column) While bound to rhodamine-labelled, biotinylated GMPCPP microtubules, SNAP-Elongator labelled with Alexa488 does not bind to tRNA-biotin-mSA2-565. (**D**) tRNA-biotin-mSA2-565 binds to (unlabelled)-SNAP-Elongator with similar affinity as to 488-tRNA. (**E**, **F**) Effect of the indicated conditions on the growth speed (**E**) and lifetime (**F**) of microtubules at the plus end imaged by TIRFM in the presence of 100 μM tRNA, 50 mM KCl and 2.5 mM $MgCl_2$. (**E**) *n*, number of microtubule-growing events analysed. *P* values for a Kruskal–Wallis test followed by Dunn's multiple comparison test are indicated. Dashed line represents an increase of ~1.4 in the speed of microtubule growth. Thick line, median; thin line, quartile. (**F**) Microtubule lifetime estimate ± error from the bootstrapped mean lifetimes (see "Methods") are indicated in the right panel. Number of microtubule-growing events analysed as in (**E**). Scale bar = 5 μm. Source data are available online for this figure.

 © The Author(s)

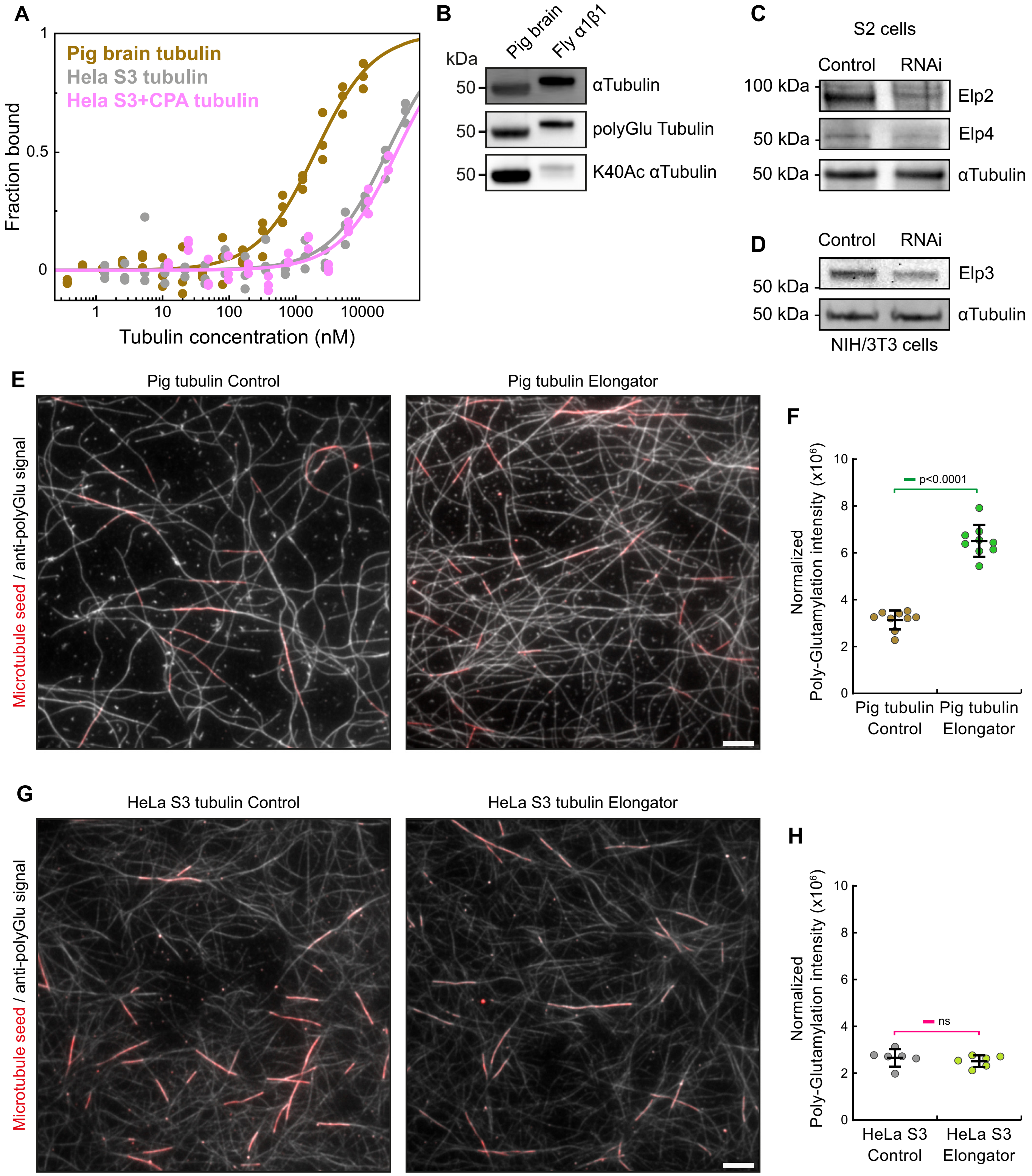
A
Pig brain tubulin
Hela S3 tubulin
Hela S3+CPA tubulin
Fraction bound
1
0.5
0
1
10
100
1000
10000
Tubulin concentration (nM)
B
Pig brain
Fly α1β1
kDa
50
αTubulin
polyGlu Tubulin
K40Ac αTubulin
C
S2 cells
Control
RNAi
100 kDa
Elp2
50 kDa
Elp4
αTubulin
D
Control
RNAi
Elp3
αTubulin
NIH/3T3 cells
E
Pig tubulin Control
Pig tubulin Elongator
Microtubule seed / anti-polyGlu signal
F
p<0.0001
Normalized Poly-Glutamylation intensity (x10^6)
10
8
6
4
2
0
Pig tubulin Control
Pig tubulin Elongator
G
HeLa S3 tubulin Control
HeLa S3 tubulin Elongator
Microtubule seed / anti-polyGlu signal
H
ns
Normalized Poly-Glutamylation intensity (x10^6)
HeLa S3 Control
HeLa S3 Elongator

© The Author(s)

Figure EV5. Elongator is a polyglutamylated tubulin polymerase.

(**A**) Measurement of the affinity between eGFP-Elp456 and αβ-tubulin heterodimers (as indicated) using microscale thermophoresis. Calculated dissociation constant (K_d) values are indicated (mean ± s.d.; $n = 3$). See also Fig. 5A,B. (**B**) Western blot for poly-Glutamylation and Lysine 40 acetylation (K40Ac) for pig brain and recombinant *Drosophila* α1β1 tubulin. (**C**, **D**) Western blots confirming the RNAi treatment in S2 cells (**C**) and NIH/3T3 cells (**D**). Note that Elp3 was not probed for in (**C**) as the Elp3 antibody does not work in lysates. (**E**, **F**) Representative field of view of microtubules labelled with fluorescently-labelled anti-polyglutamylated tubulin antibodies (gray) and HiLyte488-labelled GMPCPP stable seeds (red). Conditions as indicated. *P* value for an unpaired two-tailed t-test. Error bars are mean ± standard deviation for quantified intensities. *n* (fields of view analysed) = 9 from three independent experiments. (**G**, **H**) Quantification of total poly-glutamylated tubulin signal (see "Methods"). *P* value for an unpaired two-tailed *t* test (*P* value = 0.4547). Error bars are mean ± standard deviation for quantified intensities. *n* (fields of view analysed) = 6 from three independent experiments. Scale bars = 5 μm. Source data are available online for this figure.

 © The Author(s)
